# Phase II Trial of Sorafenib in Patients with Chemotherapy Refractory Metastatic Esophageal and Gastroesophageal (GE) Junction Cancer

**DOI:** 10.1371/journal.pone.0134731

**Published:** 2015-08-14

**Authors:** Yelena Y. Janjigian, Efsevia Vakiani, Geoffrey Y. Ku, Jessica M. Herrera, Laura H. Tang, Nancy Bouvier, Agnès Viale, Nicholas D. Socci, Marinela Capanu, Michael Berger, David H. Ilson

**Affiliations:** 1 Gastrointestinal Oncology Service, Division of Solid Tumor Oncology, Department of Medicine, Memorial Sloan Kettering Cancer Center and Weill Cornell Medical College, New York, United States of America; 2 Departments of Pathology, Memorial Sloan Kettering Cancer Center, New York, United States of America; 3 Marie-Josée & Henry R. Kravis Center for Molecular Oncology, Sloan Kettering Institute, New York, United States of America; 4 Departments of Bioinformatics, Sloan Kettering Institute, New York, United States of America; 5 Department of Epidemiology and Biostatistics, Memorial Sloan Kettering Cancer Center, New York, United States of America; Davidoff Center, ISRAEL

## Abstract

**Purpose:**

Vascular endothelial growth factor receptor (VEGFR2) directed therapies result in a modest survival benefit for patients with advanced esophageal and gastroesophageal (GE) junction cancer. Platelet-derived growth factor receptor (PDGFR) may contribute to escape from VEGFR2 inhibition. We evaluated the efficacy of sorafenib, a broad spectrum tyrosine kinase inhibitor targeting VEGFR2 and PDGFR as well as RET and RAF1, in patients with metastatic chemotherapy refractory esophageal and GE junction cancer.

**Patients and Methods:**

This phase II trial of sorafenib 400 mg twice daily enrolled chemotherapy refractory patients with metastatic esophageal and GE junction cancer with primary endpoint of progression-free survival (PFS) rate at two months. Secondary endpoints included overall survival, objective response rate and toxicity.

**Results:**

Among 34 patients, 8 week Kaplan-Meier estimated PFS was 61% (90%CI 45 to 73%). Median PFS is 3.6 months (95% CI 1.8 to 3.9 months), with median overall survival OS 9.7 months (95% CI 5.9 to 11.6 months). Grade 3 toxicities were uncommon and included hand foot skin reaction, rash, dehydration and fatigue. One patient (3%) with ongoing complete response and remains on trial for over 5 years. Whole exome sequencing of this tumor revealed mutations in many cancer-associated genes including ARID1A, PIK3CA, and TP53, and focal amplifications of HMGA2 and MET.

**Conclusion:**

Sorafenib therapy results in disease stabilization and encouraging PFS in patients with refractory esophageal and GE junction cancer.

**Trial Registration:**

ClinicalTrials.gov NCT00917462

## Introduction

The prognosis of patients with esophagogastric (EG) cancer after progression on first-line chemotherapy is poor and further cytotoxic therapy provides, at best, modest survival benefit. Molecularly targeted therapies may provide effective and potentially less toxic alternatives to chemotherapy. The Cancer Genome Atlas (TCGA) analysis revealed EG cancer as a molecularly heterogeneous disease. Aberrant activation of receptor tyrosine kinases (RTKs) through oncogene amplifications or mutations are particularly prevalent in gastroesophageal (GE) junction tumors[1], and it is likely that combinations of alterations in multiple oncogenic pathways drive EG cancer growth [2]. Therefore, simultaneous targeting of several pathways may achieve a greater therapeutic benefit.

Vascular endothelial growth factor receptor (VEGF)-2 plays a central role in tumor growth and metastasis by promoting pathologic angiogenesis [3]. Positive findings from phase III studies of the VEGFR2 inhibitors ramucirumab (a monoclonal antibody targeting VEGFR2)[4] and apatinib (a small molecule tyrosine kinase inhibitor)[5] in the second-line setting have recently intensified interest in VEGFR2 inhibition as a therapeutic strategy in esophagogastric cancer. Preclinical models suggest that upregulation of the platelet-derived growth factor (PDGF) and fibroblast growth factor (FGR) pathways provide alternate escape mechanisms to drive disease progression in setting of VEGF-VEGFR blockade [6] [7].

Sorafenib is a multi-kinase inhibitor that targets multiple RTKs including: VEGFR-2, PDGFR, RET, Flt3 and RAF1 [8,9]. On the basis of the promising biologic rationale and published clinical activity of sorafenib combined with chemotherapy in treatment naïve gastric cancer patients [10], we conducted a phase II trial to determine the single agent activity of sorafenib in chemotherapy refractory esophageal and GE junction tumors.

## Materials and Methods

### Patients

Eligible patients were at least 18 years old and had a diagnosis of Stage IV esophageal, gastroesophageal (GE) junction adenocarcinoma or squamous cell carcinoma with measurable lesions showing radiographic progressive disease on ≤2 prior chemotherapy regimens in the metastatic setting (or ≤3 prior regimens including perioperative chemotherapy or chemoradiotherapy). Other eligibility criteria included Karnofsky Performance Status (PS) of at least 60 (requires occasional assistance, but is able to care for most of personal needs) and adequate organ function.

### pERK Protein Expression

Raf activation leads to the downstream activation and phosphorylation of MEK (mitogen-activated protein kinase) and ERK (extracellular signal-regulated kinase) [11]. Therefore immunohistochemical (IHC) analysis of archival tissue for phosphorylated extracellular signal-regulated kinase (pERK) was performed. Both patients with and without pERK staining were eligible for treatment. A pathologist coded pERK expression as the percentage of positive tumor cells (scale 0%-100%) with staining intensity from 0 to 3+. Positive IHC expression was defined as ≥25% staining with intensity 2 or 3+.

### Study Design

This was a single institution, open-label, non-randomized, single-arm phase II study with primary objective of the 2 months progression free survival (PFS) rate. Patients received sorafenib 400 mg twice daily until intolerable adverse events; progressive disease or death. The study used an exact binomial single stage design 35 patients to differentiate between 2 months PFS of 50% (based on historical control[12]) and 72% with type I and II error rates of 10% each. If 22 or more patients were alive and progression free at 2 months, the regimen would be declared promising. A dose-reduction for the management of toxicity was specified in the study protocol. Briefly, on first occurrence of ≥grade 3 adverse events, sorafenib was reduced to 400 mg daily, and with second occurrence, sorafenib was reduced to 400 mg every other day.

### Study Endpoints

Efficacy endpoints included PFS, defined as the duration of time from start of treatment until progressive disease (PD) according to Response Evaluation Criteria in Solid Tumors (RECIST) or death, overall survival (OS) defined as the time from start of treatment till death or last follow up, and response defined as a best response to treatment of complete response (CR) or partial response (PR), as assessed by investigators according to RECIST criteria. Patients were assessed weekly for the first 3 weeks and monthly thereafter for toxicity based on the National Cancer Institute Common Toxicity Criteria for Adverse Events v3.0 (NCI CTCAE v3.0).

### Tumor Assessments

Tumor assessments took place at week 4, 8 and every 2 months thereafter according to RECIST, version 1.0[13] using computed tomography.

### Statistical Analysis

All patients who had received sorafenib were included in the description of baseline characteristics, efficacy and safety analysis. PFS and OS were estimated using Kaplan-Meier methodology and compared by pERK expression using log-rank test. Response rate was summarized by using binomial proportions, and exact 95% confidence intervals were provided.

### Sequencing and Bioinformatics

Available archival formalin fixed paraffin embedded (FFPE) samples of three patients with prolonged disease stabilization were analyzed alongside matched normal tissue by using an on-site 279 cancer-associated gene bait capture, next generation sequencing (NGS) assay. For the patient with complete durable response over five years, whole exome sequencing was subsequently performed in addition on frozen tumor and peripheral blood DNA. Standard sequencing and bioinformatics analysis methods were used.

### Study Conduct

The study protocol was approved by the institutional review board at Memorial Sloan Kettering Cancer Center. All patients provided written informed consent. The study was designed by senior academic authors. Sorafenib was provided by Bayer.

## Results

### Patient and Treatment

**Fig 1:**
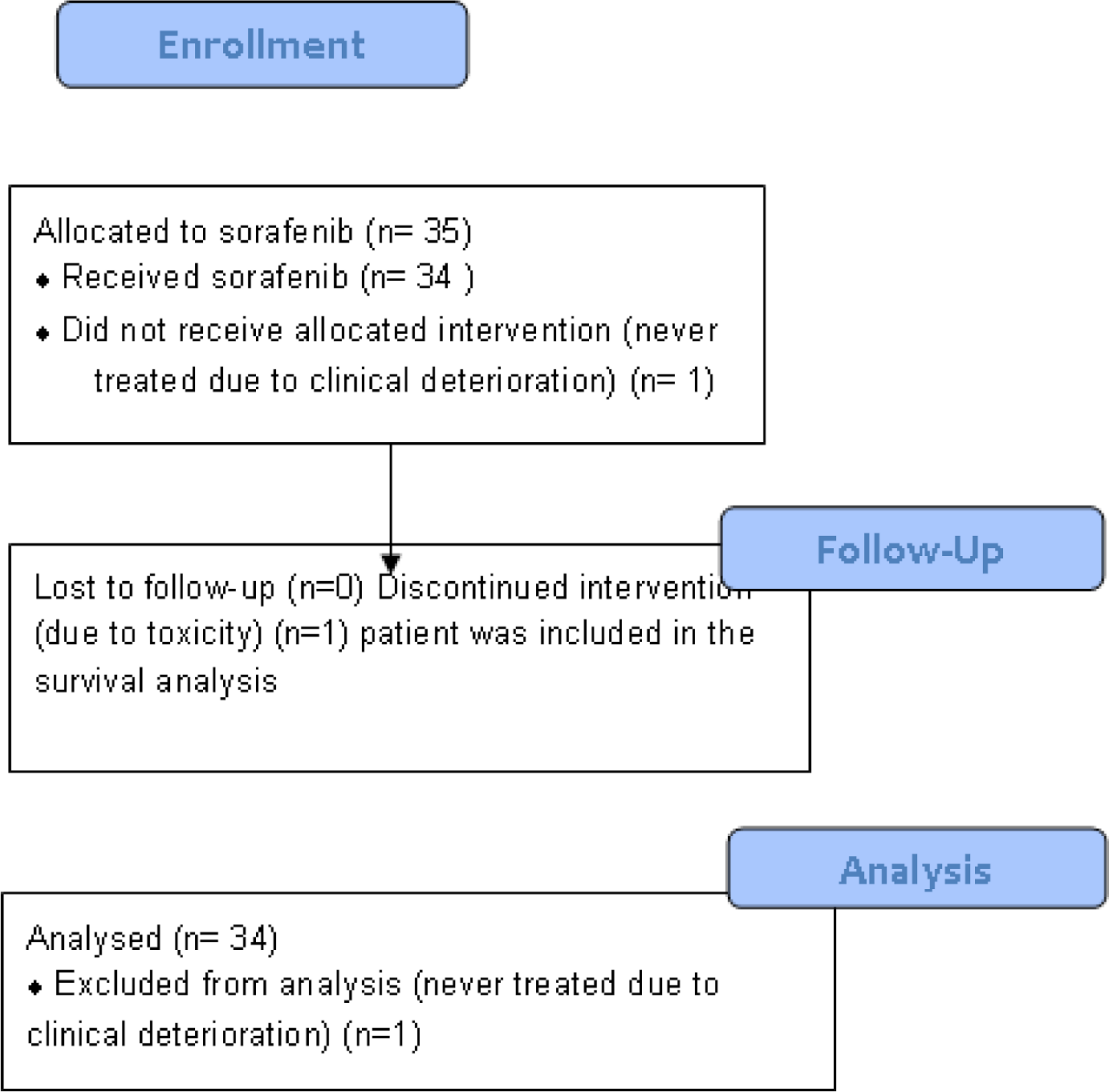
Patient enrollment and participation flow chart.

Between June 2, 2009 and August 21, 2012, 35 patients were enrolled (Fig 1). One patient was not treated due to clinical deterioration and is not evaluable. Of the 34 evaluable patients, one patient withdrew consent within the first month due to Grade 3 rash. This patient was included in PFS (censored at the patient’s last scan before sorafenib discontinuation) and OS analysis. Table 1 summarizes overall baseline patient demographics and disease characteristics of treated patients. All patients experienced disease progression or recurrence after systemic chemotherapy, with the majority of patients (62%) progressing after at least two lines of cytotoxic therapy. Adenocarcinoma was the predominant histology (85%), 68% of all enrolled patients had primary esophageal tumors, and 32% had GE junction tumors.

**Table 1:** Baseline characteristics of patients treated with sorafenib.

Patients n = 34	
**Gender, n (%)**	
**Men**	30 (88)
**Women**	4 (12)
**Age, median (range)**	61 (42 to 77)
**Baseline KPS, n (%)**	
**≥80%**	30(88)
**≤70%**	4(12)
**Prior chemotherapy, n (%)**	
**1 line**	13 (38)
**≥2 lines**	21 (62)
**Prior esophagectomy n (%)**	14 (41)
**Anatomic tumor location, n (%)**	
**Gastroesophageal junction**	11(32)
**Esophagus**	23(68)
**Histology**	
**Adenocarcinoma**	29 (85)
**Squamous Cell Carcinoma**	5(15)
**HER2 status, n (%)**	
**HER2 IHC 3+**	4 (12)
**HER2 IHC 0/1 or FISH <2**	21 (62)
**Unable to assess**	9 (26)
**p-Erk IHC, n (%)**	
**0–1+**	10 (29)
**2–3+**	22 (65)
**Unable to assess**	2 (6)

### Response and Survival

**Fig 2:**
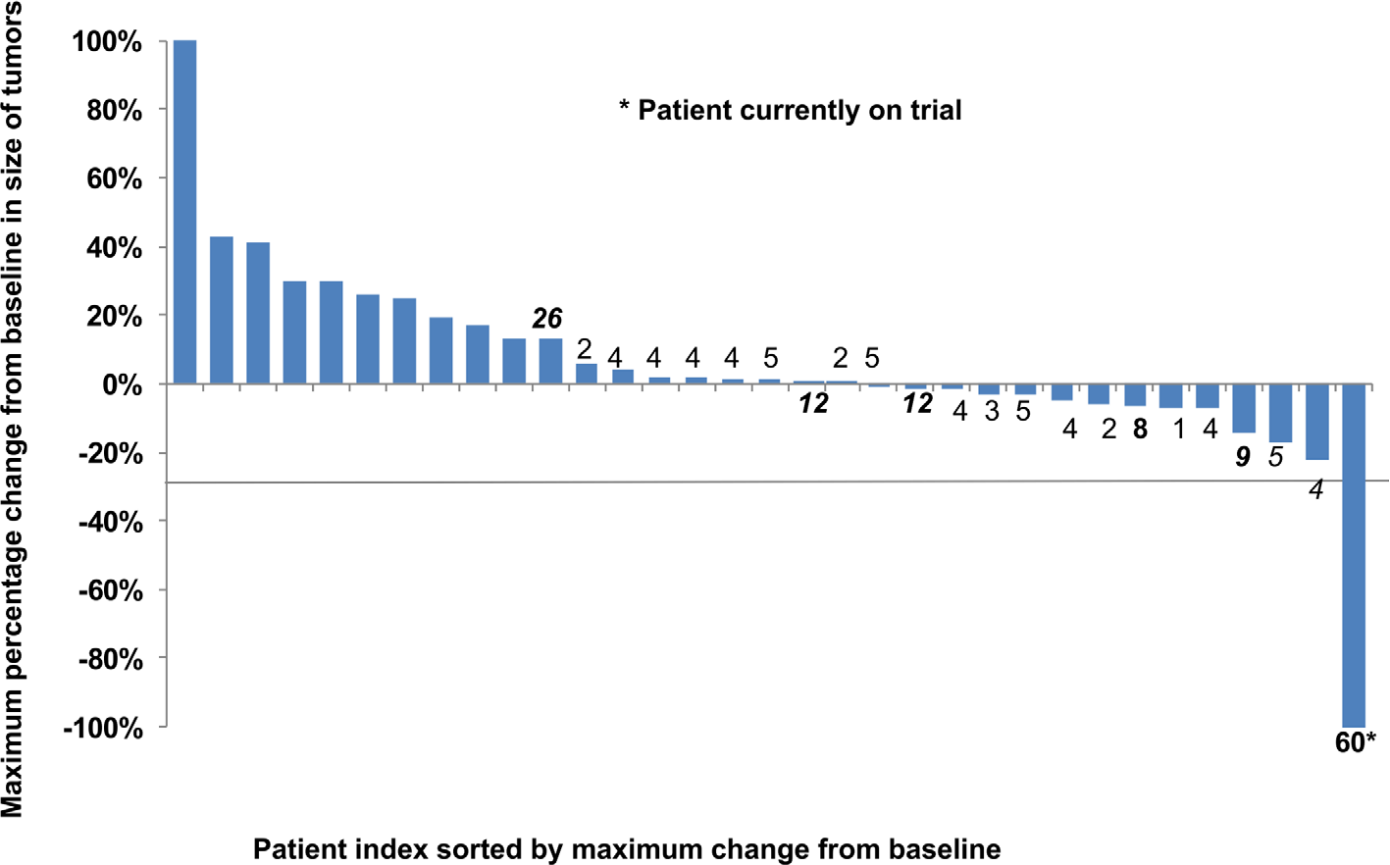
Waterfall plot showing maximum percentage change from baseline in size of tumors in patients who received sorafenib. Data available for 33 patients, one patient did not have a CT due to rapid clinical deterioration. Numbers on the bars indicate months on sorafenib, the bars without numbers indicate patients who progressed on the study rapidly within <2 months.

**Fig 3:**
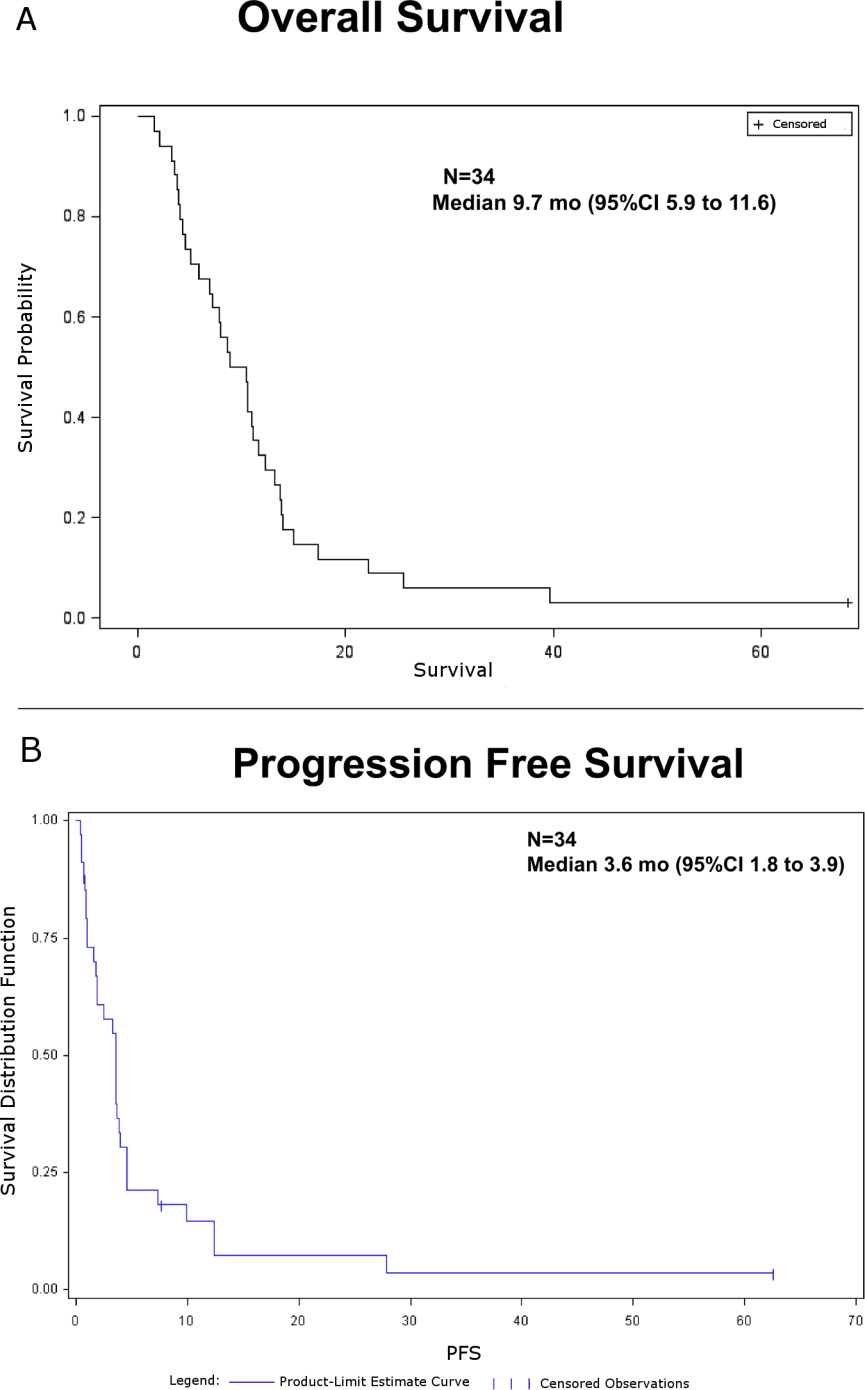
Kaplan-Meier Survival. A. Kaplan-Meier curve showing PFS in patients who received sorafenib. B. Kaplan-Meier curve showing OS in patients who received sorafenib.

Of the 34 patient evaluable for response, one (3%, 95%CI from 0% to 15%) patient with biopsy proven distant recurrence of esophageal adenocarcinoma in the neck lymph nodes within 9 weeks after combination of carboplatin and irinotecan with radiation therapy and surgery has a complete response ongoing for 5 years. Twenty of 34 patients were progression free at 2 months (Table 2). This PFS rate did not meet the pre-specified criterion of 22 of 35 patients being progression-free at 2 months. Prolonged disease stabilization of 28, 13, and 5 to 10 months was noted in one, two, and six patients, respectively. The waterfall plot in Fig 2 shows the maximum percentage change from the baseline in size of tumors in treated patients. The median PFS was 3.6 months (95%CI 1.8 to 3.9; Fig 2), 2 month Kaplan-Meier estimated PFS 61% (90%CI 45% to 73%), and the median OS 9.7 months (95%CI 5.9 to 11.6, Fig 3).

**Table 2:** Tumor assessments for all patients.

**Best Response n = 34**	**n**	**%**	**Duration in months**
**Disease progression**	**14** *	**41**	
**Stable Disease**	**19**	**56**	**(2 to 28)**
**Partial Response**	**0**	**0**	
**Complete Response**	**1**	**3**	**60+months**

*Three patients experience clinical disease progression and deterioration prior to RECIST assessment.

### pERK Protein Expression

The majority of tumors (65%) had high p-ERK expression (IHC 2+ or 3+). There was no association noted between pERK expression and outcome.

### Genomic Analysis

Adequate archival tumor samples were available from three patients treated on the study. Next generation sequencing (NGS) analysis was performed on these samples by using custom panels comprising of 279 or 341 key cancer genes. Esophageal adenocarcinoma from the complete responder harbored 18 mutations including mutations in p53, AIRD1A, PIK3CA and MET gene. In addition, this tumor had focal amplification of HMGA2 and MET genes. Adenocarcinoma from the patient with 9 months disease stabilization harbored 21 mutations, including p53, ERBB2 and ERCC1 mutations as well as focal amplifications involving ERBB2 and CCND1. Lastly, a tumor from a patient with metastatic esophageal squamous cell with 5 months stable disease revealed 8 mutations, including p53 mutation and focal amplifications of PIK3CA, SOX2, NOTCH2, and IGF1R. S1, S2 and S3 Tables provide a summary of detected mutations from targeted NGS. Whole exome analysis was performed on the esophagectomy specimen from the patient with complete response and results are shown in S4 Table.

### Toxicity

Of 34 patients, 21 required dose reduction. Fifteen patients were reduced one level to sorafenib 400mg daily, and 6 patients required two dose reductions to 400mg every other day. Most reductions were for skin toxicity, fatigue, and anorexia. One patient discontinued study therapy after one month due to intolerable Grade 3 rash. Adverse events are listed in Table 3. Grade 3/4 toxicities included hand-foot reaction (12%), vomiting (3%), fatigue (6%), dehydration (3%), hypertension (3%), and esophageal fistula (3%).

**Table 3:** Drug-Related Adverse Events Grouped by Preferred Term.

**Toxicity n = 34**	**All Grades**	**Grade 3 N %)**	**Grade 4 N (%)**
**Total Patients Treated**	**34 (100%)**		
**ALT, SGPT or AST, SGOT**	**3 (9)**		
**Dehydration**	**1 (3)**	**1 (3)**	
**Diarrhea**	**18 (53)**		
**Dry skin**	**8 (24)**		
**Fatigue (asthenia, lethargy, malaise)**	**29 (85)**	**2 (6)**	
**Fistula, GI- Esophagus**	**1 (3)**		**1 (3)**
**Hair loss/alopecia (scalp or body)**	**4 (12)**		
**Hypertension**	**2 (6)**		**1 (3)**
**Mucositis—Oral cavity**	**4 (12)**		
**Nausea**	**11 (32)**		
**Pain—Skin**	**2 (6)**		
**Rash: acne/acneiform**	**1 (3)**	**1(3)**	
**Rash: hand-foot skin reaction**	**8 (24)**	**4 (12)**	
**Vomiting**	**6 (18)**	**1 (3)**	
**Weight Loss**	**5 (15)**		

## Discussion

Twenty of 34 patients on this study were progression free at 2 months, while one patient withdrew consent within the first month of treatment. Although this PFS rate did not meet the pre-specified criterion of 22 of 35 patients being progression-free at 2 months, this trial shows evidence of clinical activity of single agent sorafenib in patients esophageal and GE junction cancer.

While comparison of results between phase II and III trials is imprecise, the PFS of 3.6 months and OS of 9.7 months demonstrated on our trial are comparable to phase III studies of single-agent irinotecan (median PFS 2.3 months with OS 8.4 months) or paclitaxel (PFS of 3.6 months with OS 9.5 months) in the second-line setting [14]. Similarly, the Eastern Cooperative Oncology Group (ECOG) study 5203, which was a phase II evaluation of first-line sorafenib with cisplatin/docetaxel chemotherapy in advanced GE junction and gastric adenocarcinoma, suggested activity comparable to the historic survival outcomes with the parent docetaxel/cisplatin/5-fluoruracil regimen [10,15]. The low incidence of hand foot syndrome (Grade 2 6%, Grade 3 12%) and diarrhea (Grade 2 6%) on this trial contrast with reported sorafenib toxicity[16] likely due to weekly visits during the initial cycles of therapy and the dose reductions.

On the other hand, other studies that have studied sorafenib in gastric cancer in the second line setting as single agent[17] or in combination with oxaliplatin[18] have failed to demonstrate benefit. In our experience, sorafenib therapy has primarily cytostatic effects and mainly serves to stabilize tumors. Therefore, patient selection in terms of functional status, the presence of symptomatic disease, and poor organ function may have contributed to the discordance between the results of this trial with the other negative studies.

The National Cancer Institute (NCI) has embarked on the Exceptional Responders Initiative (ERI) to understand the molecular underpinnings of exceptional responses to treatment [19,20]. Such analysis identified oncogenic ARAF mutations in a lung adenocarcinoma patient with near complete radiographic response for 5 years to single agent sorafenib [21]. As such, we sequenced (NGS and whole exome analysis) available archival tumor samples to investigate the genomic basis of sustained disease stabilization in two patients and one patient with outlier response on this trial [19,20]. We were not able to identify a unifying alteration predictive of benefit from sorafenib therapy in esophageal cancer, possibly because of the small numbers of patients who were studied. It may also be that circulating VEGF might be more revealing than tissue analyses. S1–S3 Tables summarize the complex profile of somatic mutations detected in the responder and the patients with prolonged disease stabilization.

Anti-VEGFR2 therapy is the first biologic strategy in an unselected patient population to impart survival benefit in chemotherapy refractory esophagogastric cancer as demonstrated by three positive phase III trials enrolling second line patients. The phase III REGARD trial was the first to demonstrate that single agent ramucirumab (VEGFR2 monoclonal antibody) improves median survival compared to placebo (median OS 5.2 *vs* 3.8 months, Median PFS 2.1 months and 1.3 months) [4]. The RAINBOW trial demonstrated improved survival and response rate for combination of ramucirumab with paclitaxel compared to placebo with paclitaxel (median OS 9.6 *vs* 7.4 months, Median PFS was 4.4 and 2.9 months) [22]. The phase III study of apatinib (small molecule tyrosine kinase inhibitor) in advanced gastric cancer once again demonstrated improvement in survival compare to placebo (median OS 6.3 *vs* 4.6 months, Median PFS 2.5 months and 1.7 months) [5]. The U. S. Food and Drug Administration approved ramucirumab as either a single agent or in combination with paclitaxel for the treatment of patients with metastatic, gastric or GE junction adenocarcinoma with disease progression after fluoropyrimidine/platinum chemotherapy.

While these data suggest an important role of angiogenesis in the progression of esophagogastric cancer, two first line trials exploring chemotherapy in combination with bevacizumab (anti-VEGF A ligand monoclonal antibody)[23] and ramucirumab[24] failed to meet their primary endpoints. These negative results suggest that, at least in a first line setting, inhibiting VEGF alone may not be sufficient and inhibition of multiple compensatory pathways such as PDGF and FGFR may be important. In a subgroup analysis, high plasma VEGF-A and low baseline tumor expression of neuropilin were suggested to be predictive of sensitivity to bevacizumab in Western patients [25]. Although we can hypothesize why these trials were negative–patient selection and the difference between esophageal vs GE junction vs gastric tumors, distinct tumor biology of intestinal vs diffuse histology, heterogeneity of gastric cancer between East and West—presently there is no biomarker to best select patients for enrollment in antiangiogenesis trials.

The success of future trials with novel molecular targets depends on biomarker-driven patient selection and tissue correlative components. To date, studies validated human epidermal growth factor receptor 2 (HER2) with trastuzumab (HER2-directed monoclonal antibody) in a first-line setting[26], and additional trials are underway investigating c-MET and PDL1 as potential biomarkers. Circulating VEGF-A levels and functional imaging looking at vascular permeability and perfusion may help select patients for future antiangiogenic therapies[27].

On the basis of these clinical data with sorafenib, we have recently completed a first line phase II trial in metastatic esophagogastric cancer of FOLFOX plus regorafenib (the next generation multi kinase inhibitor FDA approved in colorectal cancer and gastrointestinal stromal tumors [28,29]) (clinicaltrials.gov NCT01913639). The investigators in the US and Australia are exploring regorafenib in second line esophageal cancer (clinicaltrials.gov NCT02241720)[30] Given the response and protracted disease stabilization seen, we are also exploring regorafenib as adjuvant maintenance therapy in high risk resected esophageal adenocarcinoma with high disease recurrence and no current standard therapy options (clinicaltrials.gov NCT02234180).

## Supporting Information

S1 Protocol Protocol for phase II trial of sorafenib for patients with metastatic or recurrent esophageal and gastroesophageal junction cancer. (PDF)

S1 Table Summary of mutations in adenocarcinoma of sorafenib complete responder detected on next generation sequencing. (XLSX)

S2 Table Summary of mutations in adenocarcinoma from the patient with 9 months disease stabilization detected on next generation sequencing. (XLSX)

S3 Table Summary of mutations in squamous cell tumor from the patient with 5 months disease stabilization detected on next generation. (XLSX)

S4 Table Whole exome analysis of tumor from the patient with complete response on sorafenib. (XLS)

S1 TREND Checklist TREND Checklist. (PDF)
