# Supplementary material for: Phase II Trial of Sorafenib in Patients with Chemotherapy Refractory Metastatic Esophageal and Gastroesophageal (GE) Junction Cancer
Source: PLoS One. 2015 Aug 14;10(8):e0134731. doi: 10.1371/journal.pone.0134731 (PMC4537304; doi:10.1371/journal.pone.0134731)
Supplement: S1 Protocol — (PDF) [file pone.0134731.s001.pdf]

Memorial Sloan-Kettering Cancer Center
IRB Protocol

IRB#: 09-016A(3)

Phase II Trial Of Sorafenib For Patients With Metastatic Or Recurrent Esophageal And Gastroesophageal Junction Cancer

MSKCC THERAPEUTIC/DIAGNOSTIC PROTOCOL

**Principal Investigator/Department:**	David H. Ilson, MD, PhD	Department of Medicine
**Co-Principal Investigator(s)/Department:**	Manish Shah, MD	Department of Medicine
**Investigator(s)/Department:**	Ghassan Abou-Alfa, MD<br>Ki-Young Chung, MD<br>David D'Adamo, MD<br>Yelena Janjigian, MD<br>Neil Segal, MD, PhD<br>David Kelsen, MD<br>Nancy Kemeny, MD<br>Raymond Meng, MD, PhD<br>Diane Reidy-Lagunes, MD<br>Eileen O'Reilly, MD<br>Leonard Saltz, MD<br>Archie Tse, MD<br>Raymond Meng, MD	Department of Medicine
	Louis Fuqua, MD	Department of Radiology
	Laura Tang, MD	Department of Pathology
	Marinela Capanu, PhD	Department of Biostatistics

Memorial Sloan-Kettering Cancer Center
1275 York Ave. New
York, NY 10021

IRB
PB

Amended: 11/23/10

**Consenting Professional(s)/Department:**	Ghassan Abou-Alfa MD<br>Ki-Young Chung MD<br>David D'Adamo, MD<br>David Ilson, MD, PhD<br>Yelena Janjigian, MD<br>Neil Segal, MD, PhD<br>David Kelsen MD<br>Nancy Kemeny MD<br>Raymond Meng, MD, PhD<br>Eileen O'Reilly MD<br>Diane Reidy-Lagunes, MD<br>Leonard Saltz MD<br>Archie Tse, MD PhD<br>Manish Shah MD	Department of Medicine

Please Note: A Consenting Professional must have completed the mandatory Human Subjects Education and Certification Program.

TABLE OF CONTENTS

1.0 PROTOCOL SUMMARY 3

2.0 OBJECTIVES AND SCIENTIFIC AIMS 4
2.1 Primary objective 4
2.2 Secondary objectives 4

3.0 BACKGROUND AND RATIONALE 4
3.1 Esophageal cancer 4
3.2 Vascular endothelial growth factor (VEGF) and anti-angiogenic therapy 5
3.3 Angiogenesis in esophageal cancer 6
3.4 Ras/RAF/MEK/ERK pathway in esophageal cancer 7
3.5 Sorafenib 8
3.5.1 Pre-clinical rationale 8
3.5.2 Clinical experience 9
3.5.3 Renal cell carcinoma (RCC) 9
3.5.4 Hepatocellular carcinoma 10

4.0 OVERVIEW OF STUDY DESIGN/INTERVENTION 14
4.1 Design 14
4.2 Intervention 14

5.0 THERAPEUTIC/DIAGNOSTIC AGENTS 15
5.1 Sorafenib 15

CRITERIA FOR SUBJECT ELIGIBILITY 17
6.1 Subject Inclusion Critieria 17
6.2 Subject Exclusion Criteria 19

7.0 RECRUITMENT PLAN 21
7.1 Inclusion of women and minorities 21

8.0 PRETREATMENT EVALUATION 21
8.1 To be completed within four weeks prior to study entry 21
8.2 To be completed within two weeks prior to study entry 22
8.3 To be completed anytime prior to starting therapy 23

9.0 TREATMENT/INTERVENTION PLAN 23
9.1 Agent administration 23
9.2 Supportive care guidelines 24

10.0 EVALUATION DURING TREATMENT/INTERVENTION 25

11.0 TOXICITIES/SIDE EFFECTS... 28
11.1 General considerations 28
11.2 General toxicity profile.. 28
11.3 Laboratory abnormalities 30
11.4 Special warning and precautions for use 31
11.5 Dose delay/modification.. 35
11.6 Adverse events definitions 42
11.6.1 Adverse event 42
11.6.2 Serious adverse event (SAE).. 42
11.6.3 Adverse event documentation 42
11.7 Reporting of serious adverse events (SAE) 43

12.0 CRITERIA FOR THERAPEUTIC RESPONSE/OUTCOME ASSESSMENT......... 44
12.1 Measurement of effect 44
12.2 Definitions... 44
12.2.1 Measurable disease 44
12.2.2 Non-measurable disease... 45
12.2.3 Target lesions 45
12.2.4 Non-target lesions 45
12.3 Guidelines for evaluation of measurable disease.. 45
12.4 Response criteria... 46
12.4.1 Evaluation of target lesions 46
12.4.2 Evaluation of non-target lesions 47
12.4.3 Evaluation of best overall response.. 47
12.5 Confirmatory measurement/duration of response.. 48
12.5.1 Confirmation.. 48
12.5.2 Duration of overall response 48
12.5.3 Duration of stable disease 49
12.6 Progression-free survival... 49

13.0 CRITERIA FOR REMOVAL FROM STUDY 49

14.0 BIOSTATISTICS.. 50

15.0 RESEARCH PARTICIPANT REGISTRATION AND RANDOMIZATION PROCEDURES.. 51
15.1 Research participation registration.. 51

16.0 DATA MANAGEMENT ISSUES 51

IRB
PB

16.1 Quality assurance........ 52
16.2 Data and safety monitoring........ 52

17.0 PROTECTION OF HUMAN SUBJECTS........ 53
17.1 Privacy........ 54
17.2 Serious Adverse Event (SAE) Reporting........ 54

18.0 INFORMED CONSENT PROCEDURES........ 56
18.1 Research authorization........ 57

19.0 REFERENCES........ 57

20.0 APPENDICES........ 61

1.0 PROTOCOL SUMMARY AND/OR SCHEMA

Protocol title: Phase II trial of sorafenib for patients with metastatic or recurrent esophageal and gastroesophageal junction cancer

Study objectives

Primary: To evaluate the 2-month progression-free survival (PFS) of sorafenib in patients with metastatic or recurrent esophageal and gastroesophageal (GE) junction cancer. With a total of 35 patients, we have 90% power to detect an improvement in the 2-month PFS from a historical control of 50% to 70% with type I error rate of 10%.

Secondary:

a) To evaluate the overall response rate of sorafenib in the evaluable population;

b) To evaluate the tolerability and adverse event profile of sorafenib in this patient population;

c) To perform an exploratory analysis of differential response between squamous cell carcinoma and adenocarcinoma.

d) To evaluate phosphorylated extracellular signal-regulated kinase (pERK) expression in tumor tissue and correlate with tumor control.

Study population: Patients with metastatic or recurrent esophageal or GE junction squamous cell carcinoma or adenocarcinoma will be eligible. Patients will be allowed to have up to two prior chemotherapy regimens for metastatic disease or up to three prior therapies if they also received neoadjuvant/adjuvant chemotherapy or chemoradiotherapy. Prior therapy must be completed at least three weeks prior to initiation of sorafenib therapy.

Study design: Single instutition, open-label, non-randomized, single-arm phase II

Number of patients: 35

Study drugs: Sorafenib, administered orally

Amended:11/23/10

IRB PB

Dose and regimen: Sorafenib 400 mg twice daily administered continuously. Patients will continue on treatment until progression of disease. Each cycle consists of 28 days.
A CT (computerized tomography) or MRI (magnetic resonance imaging) scan of the chest and abdomen will be obtained at baseline, after the first four weeks of therapy, at eight weeks, and then every eight weeks thereafter. The same imaging modality performed at baseline (CT or MRI) will be repeated at subsequent imaging.

2.0 OBJECTIVES AND SCIENTIFIC AIMS

Primary objective:
To evaluate the 2-month progression-free survival (PFS) of sorafenib in patients with metastatic or recurrent esophageal and gastroesophageal (GE) junction cancer.

Secondary objectives:
a) To evaluate the overall response rate (partial response and complete response) of sorafenib.

b) To evaluate the tolerability and adverse event profile of sorafenib in this patient population.

c) To perform an exploratory analysis of differential response between squamous cell carcinoma and adenocarcinoma.

d) To evaluate phosphorylated extracellular signal-regulated kinase (pERK) expression in tumor tissue and correlate with tumor response.

3.0 BACKGROUND AND RATIONALE

3.1 Esophageal cancer

In 2007, there will be an estimated 15,560 new cases of esophageal cancer, with 13,940 estimated deaths.[1] The large majority of esophageal cancers are classified as squamous cell carcinoma (typically arising from the upper/middle thirds of the esophagus) or adenocarcinoma (distal esophagus or gastroesophageal (GE) junction).

While squamous cell carcinoma used to be the predominant histological subtype, this is no longer the case in the United States and Western Europe. In the U.S., the incidence of adenocarcinoma of the distal esophagus, GE junction and gastric cardia has increased 4% to 10% per year among U.S. men since 1976 so that it is now the most common histology.[2,3]

As there is no standard screening procedure for esophageal cancer, 50% of patients present with metastatic disease, where chemotherapy is the mainstay of palliative therapy. Even for patients with localized disease, surgery alone results in 5-year survival of only about 20%, with the remainder of patients developing recurrent disease.[4] The addition of neoadjuvant chemotherapy or chemoradiotherapy to surgery results in 5-year survival of no more than 25-35%.[5,6]

For patients with metastatic or recurrent disease, chemotherapy results in responses in 20-40% of patients and median survivals of only 8-10 months.[7] Prior standard chemotherapy included continuous infusion fluorouracil (5-FU) and cisplatin, the administration of which is cumbersome and requires placement of central intravenous access. Gastrointestinal and other toxicities are also substantial. Recent investigations have focused on the addition of a third chemotherapy drug (docetaxel) to the standard two-drug regimen, resulting in a modest 0.6 month improvement in overall survival, but at the expense of significant hematologic toxicity (including a 29% rate of febrile neutropenia versus 12% with 5-FU/cisplatin).[8]
Clearly, there is a need for more active and tolerable therapies.

3.2 Vascular endothelial growth factor (VEGF) and anti-angiogenic therapy

Folkman and others have provided compelling evidence linking tumor growth and metastases with angiogenesis.[9] Weidner et al. showed a statistically significant correlation between the density of microvessels in histologic specimens of human breast cancer and clinical outcome, including the incidence of metastases as well as overall and relapse-free survival.[10,11]

Of the identified angiogenic factors, vascular endothelial growth factor (VEGF) is the most potent and specific and has been identified as a crucial regulator of both normal and pathologic angiogenesis. VEGF produces a number of biologic effects, including endothelial cell mitogenesis and migration and induction of proteinases, leading to remodeling of the extracellular matrix, increased vascular permeability, and maintenance of survival for newly formed blood vessels.[12] Increased expression of VEGF has been measured in most human tumors examined to date, including tumors of the lung, breast, thyroid, gastrointestinal tract, kidney, bladder, ovary, and cervix, as well as angiosarcomas and glioblastomas.[12]

Based on this pre-clinical rationale, bevacizumab, a chimeric murine monoclonal antibody against human VEGF, has been extensively investigated in a number of solid tumor malignancies. The addition of bevacizumab to cytotoxic chemotherapy has been shown in several phase III clinical trials to increase the efficacy of chemotherapy and to improve progression free survival in patients with colorectal cancer[13], lung cancer[14] and breast cancer[15].

Our group has previously performed a multi-center phase II evaluation of bevacizumab and cisplatin/irinotecan in patients with advanced gastric and GE junction adenocarcinoma.[16] The addition of bevacizumab to cytotoxic chemotherapy significantly improved the time-to-progression (8.3 months; 95% CI, 5.5 to 9.9 months) and overall survival (12.3 months; 95% CI, 11.3 to 17.2 months) of patients, compared to a historical time-to-progression of 5 months. Therapy was well-tolerated, although a 6% incidence of gastric perforation or near-perforation and a 2% incidence of myocardial infarction were noted.

Sunitinib, an oral multi-target tyrosine kinase inhibitor (TKI) that has activity against VEGF receptor (VEGFR), has also undergone preliminary evaluation in advanced gastric cancer.[17] In data presented at ASCO 2007, in a multicenter phase II trial, 42 patients received sunitinib as second-line therapy. Of these, two patients (5%) had a PR while another 15 (36%) had SD. Median TTP was 17.1 weeks, while median OS was 50.7 weeks. Toxicities were comparable to those seen on other trials with sunitinib. Significant grade 3/4 toxicities included hand-foot reaction (10%), fatigue (10%) and anorexia (10%). Grade 3/4 hematologic toxicities included neutropenia (31%), thrombocytopenia (29%) and anemia (14%).

There are limited data to indicate what would be the expected progression free survival in patients either on supportive care alone, or receiving an inactive second line therapy. Erlotinib was inactive in our adenocarcinoma patients, and PFS was 1.6 months. Three other trials of EGFR TKI's, two limited to adenocarcinoma of the esophagus, indicated PFS of 1.9-2.0 months, consistent with our observation.[53-55] It is likely that PFS is 1.6-2 months or less in this patient population, i.e. progression occurs at the first or second month of CT scan assessment. As there are no data for best supportive care alone, these data represent the best estimate of PFS in patients treated with essentially inactive therapy.

3.3 Angiogenesis in esophageal cancer

A number of studies have evaluated the potential role of the VEGF pathway in esophageal cancer as a prognostic marker, and the potential changes in VEGF expression in patients receiving preoperative combined modality chemoradiotherapy. Studies in squamous cancers have indicated that expression of VEGF in tumors correlates with more advanced tumor stage, the presence of nodal metastasis and distant metastasis, and with a poorer survival outcome.[18]

In esophageal adenocarcinoma, increasing expression of VEGF correlates with the transition from Barrett's esophagus to high grade dysplasia, and with the transition from microinvasive to locally advanced cancer.[19,20] While several investigators have not detected a relationship between VEGF expression and outcome,[21] one series of 75 tumor samples obtained at esophagectomy did show that VEGF expression was correlated with the presence of angiolymphatic invasion, nodal metastases and survival.[22]

These pre-clinical data, coupled with the promising clinical data discussed in the previous section, suggest that angiogenesis is a viable and attractive target for therapy in advanced esophageal and GE junction cancer.

3.4 Ras/Raf/MEK/ERK pathway in esophageal cancer

The mitogen-activated protein kinase (MAPK) signaling pathway plays a critical role in transmitting proliferative signals generated by cell surface receptors and cytoplasmic signaling elements to the nucleus. Because derangements in the MAPK pathway have been linked to a number of human malignancies, many of its aberrant and critical components are potential therapeutic targets in human cancers.[23]

The MAPK pathway consists of the *ras* family of oncogenes and encoded proteins. Ras plays a central role in an intricate array of signal transduction pathways, characterized by cross-talk, feedback loops and multi-component signaling complexes.[24,25]
The Raf serine/threonine kinases are the principal effectors of Ras in the MAPK pathway. Raf activation occurs immediately downstream of membrane and cytoplasmic receptors that relay mitogenic signals.
In turn, Raf activation leads to the downstream activation and phosphorylation of MEK (mitogen-activated protein kinase kinase) and ERK (extracellular signal-regulated kinase).[26]

While aberrancies in the MAPK pathway have been well-characterized in a number of cancers, its role in the pathogenesis of esophageal cancer is unclear. However, there is some evidence to suggest that it may be active.

In one study, activation of ERK and Raf was identified in 63.3% and 60.3% of squamous esophageal cell carcinoma specimens respectively, whereas normal mucosal epithelial tissues were negative. Moreover, a close association was observed between ERK expression and the differentiation grade, with the ERK signal greater in poorly differentiated tissues than in well and moderately differentiated tissues.[27]

In addition, the MAPK pathway is thought to be activated by signaling through the epidermal growth factor receptor (EGFR), a member of the ERBB growth factor receptor tyrosine kinase family. EGFR protein expression has been reported in 30% to 70% of esophageal cancers and has been correlated with poor patient outcome and inferior response to conventional therapies.[28]

In a prior phase II evaluation of erlotinib, an anti-EGFR TKI, as second-line therapy for patients with advanced esophageal cancers performed here at MSKCC, 2 of 30 patients (7%) had a partial response.[29] Both of these patients had squamous cell histology and EGFR overexpression by immunohistochemical (IHC) staining.

In a phase II evaluation of gefitinib, another anti-EGFR oral TKI, as second-line therapy for patients with advanced esophageal cancers, 36% of tumors were found to express phosphorylated ERK (pERK) by IHC staining.[30] However, there did not appear to be a difference in response to gefitinib therapy between pERK-positive and pERK-negative patients.

3.5 Sorafenib

3.5.1 Pre-clinical rationale

Sorafenib (BAY 43-9006) is an oral multi-kinase inhibitor with effects on tumor proliferation and tumor angiogenesis.[31] It has inhibitory activity against the tyrosine kinases for VEGFR and platelet-derived growth factor (PDGF) receptor, as well as Flt-3 and c-Kit.[31] In addition, it was also initially selected on the basis of inhibitory activity against the serine/threonine kinases Raf-1 and wild-type B-Raf, which are pivotal components of the Ras/Raf/MEK/ERK signaling pathway.[32]

In preclinical studies, sorafenib demonstrated broad-spectrum anti-tumor activity by inducing complete tumor stasis and inhibition of tumor angiogenesis in a variety of tumor types,[31] including renal cell carcinoma (RCC), colon cancer, non-small cell lung cancer, hepatocellular cancer, pancreatic cancer, ovarian cancer and breast cancer cell lines.[33]

IRB
PB

3.5.2 Clinical experience

The safety and clinical activity of sorafenib, either alone[34-37] or in combination with chemotherapy[38,39], has been examined in a series of phase I studies conducted in patients with solid tumors.

The clinical activity of sorafenib in metastatic RCC was first demonstrated in a phase II randomized discontinuation trial in patients with metastatic RCC.[40] In this study, all patients were treated with sorafenib 400 mg BID and were assessed at 12 weeks. Patients with tumor shrinkage ≥25% continued on open-label sorafenib and patients with tumor growth ≥25% were taken off-study. Patients with tumor shrinkage or tumor growth < 25% were randomized to receive either sorafenib or placebo. At 12 weeks post-randomization, 50% of the sorafenib patients were progression-free, compared to only 18% of patients receiving placebo. Sorafenib significantly prolonged median progression-free survival from randomization versus placebo (24 versus 6 weeks; p=0.009).

The most common adverse events were fatigue (73% of patients), rash/desquamation (66%), hand-foot skin reaction (62%), pain (58%) and diarrhea (58%). The majority of these events were grade 1/2 in severity. Nine patients discontinued therapy because of toxicity. The most common grade 3/4 adverse event was hypertension, which was observed in 31% of patients; antihypertensive therapy was initiated in 46% of patients.

3.5.3 Renal cell carcinoma (RCC)

Based on the promising phase II results, a multicenter, double-blind randomized phase III evaluation of sorafenib 400 mg BID versus placebo was undertaken.[41] The primary endpoint of the trial was overall survival (OS), with progression-free survival (PFS) as a secondary endpoint. Patients with advanced RCC with good performance status following one prior systemic therapy were randomized to either sorafenib or blinded placebo with best supportive care.

From November 2003 to March 2005, 903 patients were randomized. In January 2005, a per-protocol interim analysis of PFS demonstrated a statistically significant benefit for sorafenib over placebo. As such, the study was unblinded in May 2005 and sorafenib was offered to patients who were initially randomized to receive placebo.

At the time of cross-over, an interim analysis of OS was conducted. Median OS in the placebo group was 14.7 months and had not been reached in the sorafenib group (hazard ratio 0.72; 95% CI, 0.54 to 0.94, p=0.02). The threshold for statistical significance for this interim analysis was not reached (p< 0.0005).
Final analysis of OS was reported at ASCO 2007.[42] Median OS in the sorafenib group was 17.8 months and 15.2 months in the placebo group (hazard ratio 0.88, p=0.15). For this study, however, OS was likely confounded by the fact that approximately half of the placebo patients crossed over at the time of unblinding to receive sorafenib. Indeed, OS analysis with placebo patients censored at the time of crossover indicated that sorafenib treated patients had a statistically significant 22% reduction in mortality risk compared to placebo patients (OS 17.8 months versus 14.3 months; hazard ratio 0.78, p=0.03).

Median PFS was 5.5 months for sorafenib versus 2.8 months for placebo (p<0.0001). Sorafenib was associated with a reduction of 56% in the independently-assessed risk of progression (hazard ratio 0.44; 95% CI, 0.35 to 0.55).
At the May 2005 cutoff, among the 451 evaluable patients in the sorafenib group, 1 patient had a complete response (<1%), 43 had a partial response (10%) and 333 had stable disease (74%). Among 452 patients in the placebo group, no patient had a complete response, 8 had a partial response (2%) and 239 had (53%) stable disease. Significantly more patients in the sorafenib group had a partial response or stable disease (p<0.001).

In December 2005, the U.S. Food and Drug Administration (FDA) approved sorafenib for the treatment of patients with advanced RCC.

3.5.4 Hepatocellular carcinoma

A multicenter, randomized, placebo-controlled, double-blind phase III trial of sorafenib in advanced hepatocellular carcinoma (HCC) was reported at ASCO 2007.[43] In the SHARP study, 602 patients with advanced HCC, Child-Pugh class A, with no prior systemic therapy were randomized to receive either sorafenib 400 mg BID (n=299) or placebo (n=303). In February 2007, based on a planned interim analysis of OS, the study group recommended unblinding and closure of the study. Median OS in the sorafenib group was 46.3 weeks versus 34.4 weeks in the placebo-treated group. This represented a 44% increase in median OS (hazard ratio 0.69, p=0.001). Median PFS was also significantly increased in the sorafenib group (24 weeks) versus the placebo group (12.3 weeks) (hazard ratio 0.58, p=0.00001).

IRB PB

In a prior open-label, single-arm, multicenter phase II evaluation of sorafenib in HCC led by our group, Abou-Alfa et al. demonstrated that higher tumor expression of pERK as determined by IHC was correlated with significantly improved time-to-progression compared to patients whose tumors had lower levels of pERK expression (p=0.0003).[44]

3.5.5 Risk of bleeding complications and squamous cell histology on VEGF-targeted therapy trials.

Data from squamous cancer lung cancer trials have indicated potential fatal bleeding complications with the use of the VEGF targeted agent bevacizumab (48,49). However, the registration trial in NCSLC reported a small but finite rate of fatal pulmonary hemorrhage in non squamous cancers. Recent non small cell lung cancer studies have also included squamous cancer histology in trials of VEGF-targeted agents. Data from recent and ongoing trials presented at the recent ASCO 2008 meeting indicate that VEGF targeted agents, including bevacizumab and sunitinib, were safe and tolerable in squamous cancer of the head and neck. None of these trials reported bleeding complications that led to discontinuation of the trials.

Two recent reviews discussed the historical experience of bevacizumab in NSCLC, and recent trials of VEGF-tarted agents (48,49). The initial randomized phase II trial combining bevacizumab with carboplatin and paclitaxel in 99 patients with NSCLC reported an increased incidence of fatal pulmonary hemorrhage associated with squamous cell histology; 6 events occurred in 62 patients receiving bevacizumab, five were fatal. Six patients had centrally located tumors, 5 had cavitation, and 4 were squamous cell. This observation led to subsequent exclusion of squamous cell patients on the pivotal phase III trial in non squamous cell patients, and patients with prior hemoptysis were also excluded. This trial showed a response rate, time to progression and overall survival benefit for the addition of bevacizumab to chemotherapy. However, pulmonary hemorrhage was more common on the bevacizumab arm (1.9% in 434 patients versus 0.2% in 444 patients), including 5 cases of fatal pulmonary hemorrhage in 1.2% on the bevacizumab arm.

Other trials reviewed included a trial combining gefitinib with the VEGF-targeted agent sorafenib, in NSLC indicating no dose limiting toxicities in 32 patients. A phase II trial of sorafenib in NSLC reported one one pulmonary hemorrhage (fatal) in 52 patients, in a patient with squamous cancer with a central, cavitary lesion which occurred one month after sorafenib therapy in the setting of palliative radiotherapy. Sunitinib, another VEGF-tyrosine kinase inhibitor, was evaluated in a phase II trial in 63 patients, and reported one fatal

pulmonary hemorrhage. Phase III evaluation of both of these agents in NSLC is ongoing in combination with chemotherapy. Cedaranib, another VEGF-tyrosine kinase inhibitor, is in ongoing phase II evaluation in combination with carboplatin and paclitaxel, and no pulmonary hemorrhages have been reported in 20 patients to date.

In a recently reported phase II trial, 22 patients were treated with sunitinib in recurrent or metastatic head and neck cancer (50). One partial response was observed, but the trial was closed due to failure to achieve efficacy endpoints. Vascular complications included 2 pulmonary hemorrhages, one primary tumor bleed, and 2 gastrointestinal bleeds. The authors reported the agent as well tolerated and again the trial was not closed or amended for toxicity concerns.

In a recent phase II trial, 25 patients with recurrent or metastatic head and neck cancer received pemetrexed in combination with bevacizumab (51). 2 grade 3 tumor bleeds were observed, and 1 from a gastric ulcer, and one patient had a fatal tracheal tumor bleed that the authors claimed was not treatment related but caused by aggressive suctioning. The trial indicated activity and accrual continues.

A recent report updated results on a trial treating 18 patients with recurrent or metastatic head and neck cancer with the combination of cetuximab and bevacizumab (52). Responses were seen in 4 patients, and no bleeding events were observed. This trial also continues to accrue patients.

Although bleeding is a potential complication of anti VEGF therapy, all of the above trials continued or are continuing patient accrual as the events were manageable. Because GI bleeding has even been reported for EGFR tyrosine kinase inhibitors, we will require that patients be treated with antacid therapy as we did on our prior phase II trial of erlotinib. There is therefore not an expectation, based on the above results, that bleeding complications will preclude safe administration of Sorafenib on this trial in squamous cancers of the esophagus.

3.5.6 Rationale for the Current Study

There is a clear rationale to study more than one VEGF targeted agent in esophageal cancer, as has been indicated by experience in other solid tumor malignancies and other targeted agents. There have been inconsistent benefits for targeted agents within the spectrum of GI

malignancies in addition to other solid tumors, underscoring the need to evaluate each new promising agent independently in each tumor type.

For example, in GI malignancies, the EGFR tyrosine kinase inhibitors erlotinib and gefitinib have been inactive as single agents in adenocarcinoma of the colon, stomach, and esophagus, and trials combining these agents with chemotherapy in non small cell lung cancer have failed. However in pancreatic cancer, erlotinib plus chemotherapy with gemcitabine yielded positive results, including a progression free and overall survival benefit. This is one of the first studies to show a benefit for an EGFR TKI combined with chemotherapy; indeed this is the only phase III trial in pancreatic cancer that has yielded a survival benefit compared to gemcitabine alone. On the other hand, the EGFR targeted antibody cetuximab failed when combined with chemotherapy in pancreatic cancer. This is in contrast to the single agent activity for cetuximab, and even greater activity when combined with chemotherapy, in colorectal cancer; cetuximab also has some limited single agent activity in esophagogastric adenocarcinoma.

Similarly, bevacizumab in combination with chemotherapy consistently improves response rates, progression free, and often overall survival in trials in colorectal, breast, and non small cell lung cancer; however, in pancreatic cancer, no benefit of any sort was seen for bevacizumab in combination with chemotherapy. Lastly, in breast cancer, whereas cetuximab, erlotinib, and gefitinib have all failed to yield any signal of activity, the broader spectrum HER tyrosine kinase inhibitor lapatinib, which inhibits HER-1 (EGFR) and HER-2 tyrosine kinases, has single agent activity and benefits chemotherapy effectiveness as well. Lapatinib however has failed in recent trials in esophagogastric adenocarcinoma.

It is clear that each new targeted agent, with individual variation in what specific targets are actually affected, needs to be evaluated in each solid tumor. Sorafenib is of particular interest in GI malignancies given its recent demonstration as the only agent shown to improve survival in hepatocellular cancer. Ongoing phase II trials of sorafenib in neuroendocrine carcinoma also indicate significant responses and protracted disease stabilization, in the setting of a modest toxicity profile. Sorafenib, which inhibits the tyrosine kinases associated with the VEGF receptor and the PDGF receptor, also targets the serine/threonine kinases Raf-1 and B-Raf. Its favorable toxicity profile may make this agent a promising partner with combination chemotherapy and radiation therapy and further development of this agent in gastrointestinal malignancies is clearly indicated.

4.0 OVERVIEW OF STUDY DESIGN/INTERVENTION

4.1 Design

This is a single institution, open-label, non-randomized, phase II evaluation of sorafenib 400 mg BID in patients with metastatic or recurrent esophageal or gastroesophageal (GE) junction squamous cell carcinoma or adenocarcinoma.

Patients may have received up to two prior chemotherapy regimens for their metastatic disease or up to three prior regimens if they received neoadjuvant/adjuvant chemotherapy or chemoradiotherapy. All patients must be able to provide informed consent prior to enrollment.

See Section 10 for treatment schema.

4.2 Intervention

35 patients will be enrolled on this clinical trial. Patients will receive sorafenib 400 mg BID continuously until disease progression, unacceptable toxicity or serious intercurrent illness develops or if patient consent is withdrawn.

The primary endpoint will be 2-month progression-free survival (PFS). With a total of 35 patients, we have 90% power to detect an improvement in the 2-month PFS from a historical control of 50% to 70% with type I error rate of 10%. Secondary endpoints include the response rate to sorafenib and its toxicity. Our basis for PFS estimate in this patient population comes from our prior phase II trial of erlotinib in esophageal cancer (which had essentially no activity in adenocarcinoma of the esophagus), and nearly identical PFS results reported for this patient population treated with either erlotinib or gefitinib (1.6-2.0 months PFS in all studies) (54-56).

Patients must have measurable disease and will undergo a computerized tomography (CT) or magnetic resonance imaging (MRI) scan of the chest and abdomen at baseline, after the first four weeks of therapy, at eight weeks, and every eight weeks thereafter, with a scheduling window of up to one to fourteen (1-14) days. Response assessment will be by RECIST criteria.[45] The same imaging modality performed at baseline (CT or MRI) will be repeated at subsequent imaging.

Therapy will be administered in the outpatient setting, with each cycle consisting of 28 days of continuous therapy. The cycle start date will coincide with the physician visit date. A study diary will be completed by patients to ensure compliance with the study drug (see Appendix A).

Patients will be required to take antacid therapy to reduce the potential for gastrointestinal bleeding. Allowable agents include but are not limited to ranitidine, omeprazole, carafate or similar agents.

5.0 THERAPEUTIC/DIAGNOSTIC AGENTS

Sorafenib

Other names: BAY 43-9006, Nexavar®

Classification: Kinase inhibitor

Molecular weight: Sorafenib tosylate: 637 Daltons; sorafenib (free base): 465 Daltons

Mode of action: Sorafenib is a potent inhibitor of VEGF-R, PDFG-R, c-raf, wild-type and mutant b-raf, Flt3, c-KIT and p38α, a member of the MAPK family.

Approximate Solubility: 0.19 mg/100 mL in 0.1 N HCl, 453 mg/100 mL in ethanol, 2971 mg/100mL in PEG 400

How supplied: BAY 43-9006 sorafenib 200 mg is supplied as round, biconvex, red-film-coated tablets, debossed with the 'Bayer cross' on one side and '200' on the other side. The tablets contain BAY 43-9006 tosylate equivalent to 200 mg of the free base BAY 43-9006, and the excipients croscarmellose sodium, microcrystalline cellulose, hypromellose, sodium lauryl sulfate, and magnesium stearate. The film-coat consists of hypromellose, polyethylene glycol, titanium dioxide and red iron oxide. The film coating has no effect on the rate of release of the active BAY 43-9006 tosylate.

The study drug can be supplied as BAY 43-9006 sorafenib 200 mg

IRB PB

commercial tablets in bottles of 140 tablets with a product identification label affixed or as commercial Nexavar in bottles of 120 tablets.

Storage: Store at controlled room temperature (15 -25 ^{o}C). Storage conditions should not exceed 25 ^{o}C.

Stability: Stability studies are ongoing. The current shelf life is 24 months when stored at controlled room temperature.

Route of Administration: Orally

Sorafenib is supplied as 200 mg tablets. The dose of sorafenib is 400 mg orally twice daily for 28 days (continuously). There is no planned interruption between treatment cycles. The cycle start date will coincide with the physician visit date. Because of the potential need for physician visit scheduling to vary (due to both physician and patient issues), to avoid violation of, and deviation from, the protocol, these visits may vary by up to one to fourteen (1-14) days.

Sorafenib will be taken in the outpatient setting on Days 1-28 of each 28-day cycle. Patients should swallow the tablet whole with approximately 250 ml (8 oz.) of water each morning and evening,. Sorafenib tablets may be crushed and administered via a feeding tube if necessary.

Sorafenib should be taken without food (1 hour before or 2 hours after eating). If it is taken with meals, patients will be instructed to take sorafenib with a low- to moderate-fat diet. A high fat meal causes a decrease in sorafenib absorption (a 29% decrease in sorafenib area-under-the-curve (AUC) concentration and a 38% decrease in sorafenib maximum concentration (C_{max}) value).

Dose modifications for sorafenib are detailed in Section 11.5. Patients will be taken off sorafenib at disease progression.

Drug metabolism interactions: Sorafenib is primarily metabolized by two parallel pathways: CYP3A4-mediated oxidation and UGT1A9-mediated glucoronidation.[33] Pharmacokinetics analysis from previous clinical trials have not demonstrated any significant drug interactions when sorafenib was administered together with ketoconazole, a potent inhibitor of CYP3A4.

Therefore, it is thought that sorafenib may be safely coadministered with substrates and inhibitors of CYP3A4, such as ketoconazole, itraconazole, fluconazole and ritonavir.[33] There is no clinical information on the effect of CYP3A4 inducers on the pharmacokinetics of sorafenib.[33] Inducers of CYP3A4 activity (e.g. rifampin, *Hypericum perforatum* also known as St. John's wort, phenytoin, carbamazepine, phenobarbital, and dexamethasone) may increase metabolism of sorafenib and thus decrease sorafenib plasma concentrations.

Concomitant administration of midazolam, dextromethorphan and omeprazole, which are substrates of cytochromes CYP3A4, CYP2D6 and CYP2C19, respectively, following four weeks of sorafenib administration did not significantly alter the exposure of these agents.[33] This indicates that sorafenib is neither an inhibitor nor a clinically meaningful inducer of these cytochrome P450 isoenzymes.

Sorafenib is also known to inhibit CYP2C9 *in vitro*. Substrates of CYP2C9 include warfarin and digoxin. However, the previous phase III evaluation of sorafenib versus placebo for advanced renal cell carcinoma did not reveal a significant difference in PT/INR between both groups, suggesting that sorafenib may not have *in vivo* inhibitory activity against CYP2C9.[33] Nevertheless, routine monitoring of the PT/INR for patients on coumadin therapy is warranted.

Availability: Following submission and approval of the required regulatory documents, a supply of sorafenib may be ordered from Bayer Pharmaceuticals.

6.0 Criteria for subject eligibility

6.1 Subject Inclusion Criteria

To be included in the study, patients must fulfill all of the following inclusion criteria and not meet any of the exclusion criteria. Patients who give written informed consent prior to any study specific screening procedures proceed with the understanding that the patient has the right to withdraw from the study at any time, without prejudice.

- Patients must have histologically proven or cytologically confirmed esophageal cancer (squamous cell carcinoma or adenocarcinoma) or adenocarcinoma of the gastroesophageal (GE) junction documented at MSKCC.

- Metastatic disease measurable on a CT or MRI scan. Locally recurrent disease that is not amenable to potentially curative surgery or radiation therapy is also allowed. Lesions must be ≥10 mm in size. The primary tumor is not considered measurable disease. Recurrent or metastatic lesions within a prior radiation field are acceptable as long as disease has progressed in the radiation field by RECIST criteria. See Section 12.2 for definition of measurable lesions. The same imaging modality performed at baseline (CT or MRI) will be repeated at subsequent imaging.

- Patients are allowed to have a maximum of two prior chemotherapy regimens for metastatic disease. Patients are allowed to have a maximum of three prior regimens if they also previously received neoadjuvant/adjuvant chemotherapy or chemoradiotherapy. The last treatment must have been administered >3 weeks prior to initiation of therapy with sorafenib.

- Pathologic tissue must be available for immunohistochemistry (IHC) staining for phosphorylated extracellular signal-regulated kinase (pERK). Both patients with and without pERK staining are eligible for treatment. Submission of slides and IHC testing for pERK may be done during the course of therapy and are not required prior to protocol therapy.

- Age ≥18 years.

- Life expectancy >3 months.

- Karnofsky performance status ≥60%.

- At baseline, patients must have normal organ and marrow function as defined:

Adequate bone marrow, liver and renal function as assessed by the following:

Hemoglobin ≥ 9.0 g/dl

Absolute neutrophil count (ANC) ≥1,500/mm^3

Platelet count ≥ 100,000/mm^3

Total bilirubin ≤ 1.5 times ULN

ALT and AST ≤ 2.5 times the ULN (≤ 5 x ULN for patients with liver involvement)

Creatinine ≤ 1.5 times ULN

- Patients must have the ability to comprehend and willingness to sign an informed consent document.

6.2 Subject Exclusion Criteria

- Patients who have not recovered from adverse events related to therapy administered >3 weeks earlier. This does not include hemoglobin or other hematologic or laboratory criteria, as long as eligibility are met as outlined in 6.1.

- Patients may not be receiving any other investigational agents.

- Prior therapy with sorafenib-related compounds or compounds of similar biologic or chemical components, including compounds targeting VEGF, VEGF-R or RAF kinase.

- Uncontrolled intercurrent illness including, but not limited to, ongoing or active infection, greater than New York Heart Association (NYHA) Class II congestive heart failure, unstable or new onset angina pectoris or myocardial infarction within the past six months, unstable arrhythmia, or psychiatric illness/social situation, e.g. severe schizophrenia, that would limit compliance with study requirements. Patients with chronic arrhythmias, such as paroxysmal atrial fibrillation or paroxysmal supraventricular tachycardia, are eligible.

- Uncontrolled hypertension, defined as systolic blood pressure >150 mmHg or diastolic blood pressure >90 mmHg, despite optimal medical management.

- Thrombotic or embolic event, including cerebrovascular accident or transient ischemic attack within the past six months. Patients with prior deep vein thromboses or pulmonary emboli on a stable anticoagulation regimen will be eligible for enrollment.

- Any factor that would significantly interfere with the inability to consume or absorb an oral medication, e.g. severe nausea/vomiting not controlled by an aggressive anti-emetic regimen, grade 3/4 dysphagia, extensive small bowel resection or active inflammatory bowel disease leading to chronic malabsorption. Patients with enteral feeding tubes are eligible as sorafenib can be crushed.

- Known human immunodeficiency virus (HIV) infection or chronic Hepatitis B or C infection.

- Patients with any other concurrent disease which, in the judgment of the investigator, would make the patient inappropriate for participation in the study.

- Patients who are taking St. John's wort or rifampin (as there may be drug-drug interactions with sorafenib; see Section 5.0 and 11.4).

- Patients with known brain metastases or meningeal carcinomatosis are excluded. Patients with neurological symptoms must undergo a CT scan/MRI of the brain to exclude brain metastasis.

- Pregnant women are excluded because sorafenib has the potential for teratogenic or abortifacient effects. Female patients must either not be of childbearing potential or must have a negative pregnancy test ≤ 7 days prior to treatment. Female patients are considered not of childbearing potential if they are surgically sterile (they have undergone a hysterectomy, bilateral tubal ligation or bilateral oophorectomy) or if they are post-menopausal. Men must use effective birth control if their partners are of child-bearing potential.

- No other malignancy is allowed except for adequately treated carcinoma in-situ of the cervix, superficial transitional cell carcinoma of the bladder or basal/squamous cell skin cancer. Other cancers are permissible if the patient has been disease free for ≥3 years.

- Pulmonary hemorrhage/bleeding event ≥ CTCAE Grade 2 within 4 weeks of first dose of study drug.
- Any other hemorrhage/bleeding event ≥ CTCAE Grade 3 within 4 weeks of first dose of study drug.
- Serious non-healing wound, ulcer, or bone fracture.

- Evidence or history of bleeding diathesis or coagulopathy
- Major surgery, open biopsy or significant traumatic injury within 4 weeks of first study drug.
- Known or suspected allergy to sorafenib or any agent given in the course of this trial.

7.0 RECRUITMENT PLAN

This will be a single institution, phase II study. Patients with metastatic or recurrent esophageal and gastroesophageal (GE) junction cancer who are eligible will be identified for enrollment. No additional measures, e.g. advertisement, payment to patients, will be employed to recruit patients.

Inclusion of women and minorities

The investigators take due notice of the NIH policy concerning inclusion of women and minorities in clinical research populations. There will be no limitation with regards to race or gender.

Our institutional demographics for accrual of patients on esophageal and GE junction cancer trials reflect the national incidence of this disease. 10-15% of our patients have been women. African-American males (almost exclusively with esophageal squamous cell carcinoma) comprise 3-5% of patients treated on protocol. Given that our protocol accrual closely reflects the national incidence of this disease, no specific strategy will be undertaken to recruit women or persons of color on this trial.

This protocol/project does not include children because the number of children with esophageal and GE junction cancer is very small and because the majority are already accessed by a nation-wide pediatric cancer research network. This statement is based on exclusion 4b of the NIH Policy and Guidelines on the Inclusion of Children as Participants in Research Involving Human Subjects.

8.0 PRETREATMENT EVALUATION

To be completed within four weeks prior to study entry:

- A 12-lead electrocardiogram (ECG).

To be completed within two weeks prior to study entry:

- History and physical exam, including blood pressure, vitals, height, weight and performance status.

- Documentation of all measurable disease with a baseline computerized tomography (CT) or magnetic resonance imaging (MRI) scan of the chest and abdomen. All measurable lesions used to define response must be ≥10 mm in size. The primary tumor is excluded. Lesions within prior radiation fields are permitted as long as these lesions have documented progression in size.

- White blood cell (WBC) count, platelet count with automated differential.

- Comprehensive metabolic panel, including electrolytes, glucose, blood urea nitrogen (BUN), creatinine, lactate dehydrogenase (LDH), albumin, aspartate aminotransferase (AST or SGOT), alanine aminotransferase (ALT or SGPT), alkaline phosphatase, total bilirubin, total protein, prothrombin time (PT), international normalized ratio (INR) and partial thromboplastin time (PTT).

- Amylase/lipase.

- Serum pregnancy test, for all women of childbearing potential.
  Prior to study enrollment, women of childbearing potential (WOCP) must be advised of the importance of avoiding pregnancy during trial participation and the potential risk factors of an unintentional pregnancy. In addition, men enrolled on this study should understand the risk to any partner of childbearing potential and should practice an effective method of birth control.

  All WOCP must have a negative pregnancy test ≤7 days prior to starting therapy.

  In addition, all WOCP should be instructed to contact the Investigator immediately if they suspect they might be pregnant (e.g. late or missed period) at any time during study participation.

To be completed anytime prior to starting therapy

- Histological confirmation of metastatic esophageal or GE junction squamous cell carcinoma or adenocarcinoma prior to study enrollment. Patients without histological confirmation of recurrence or metastasis will undergo a biopsy to confirm recurrence, unless the risk of such a procedure outweighs the benefits of confirming recurrent disease.

9.0 TREATMENT/INTERVENTION PLAN

9.1 Agent administration

Treatment will be administered on an outpatient basis. Reported adverse events and potential risks are described in Section 11. Appropriate dose modifications are described in Section 11.5. Criteria for discontinuing treatment are listed in Section 13.

Sorafenib is supplied as 200 mg tablets. The starting dose of sorafenib is 400 mg orally twice daily for 28 days (continuously). There is no planned interruption between treatment cycles. The cycle start date will coincide with the physician visit date. Because of the potential need for physician visit scheduling to vary (due to both physician and patient issues), to avoid violation of, and deviation from, the protocol, physician visits may vary by up to one to fourteen (1-14) days from a strict 28 day schedule.

Sorafenib will be taken on Days 1-28 of each 28-day cycle. Patients shall swallow the tablet whole with approximately 250 ml (8 oz.) of water each morning and evening. Tablets may be crushed and administered via a feeding tube.

Sorafenib may be taken with or without food. If it is taken with meals, patients will be instructed to take sorafenib with a low- to moderate-fat diet.

A study diary will be completed by patients to ensure compliance with sorafenib (see Appendix A).

Patients will be required to take antacid therapy to reduce the potential for gastrointestinal bleeding. Allowable agents include but are not limited to ranitidine, omeprazole, carafate or similar agents.

9.2 Supportive care guidelines

- Patients should receive full supportive care, including transfusions of blood and blood products, antibiotics and anti-emetics. The reasons for treatment, dosage and dates of all supportive treatments should be recorded.
- Use of erythropoietin alfa (Epoetin alfa, Procrit®) and/or darbepoetin (Aranesp®) is permitted at the discretion of the treating physician based on standard MSKCC criteria.
- Patients who develop hand-foot reaction may receive topical emollients (such as Aquaphor®) or topical steroid or anti-histamine ointments, if appropriate.

10.0 EVALUATION DURING TREATMENT/INTERVENTION

	Pre-study[I]	Cycle 1[J]				Cycle 2 & thereafter[J]				Off-study
		Wk 1	Wk 2	Wk 3	Wk 4	Wk 1	Wk 2	Wk 3	Wk 4	
Sorafenib twice daily		Continuous								
Informed consent	X									
Demographics	X									
Medical history	X									X
Physical exam [K]	X	X	X	X		X				X
Collect sorafenib diary						X				X
Vitals [K]	X	X	X	X		X				X
Blood pressure measurement[A K]	X	X	X	X		X				X
Height	X									
Weight [K]	X	X	X	X		X				X
Performance status [K]	X	X	X	X		X				X
Concomitant medications [K]	X	X				X				X
CBC with diff, plts [K]	X	X	X	X		X		X[B]		X
Comprehensive metabolic panel[C K]	X	X		X		X				X
Amylase/lipase[D]	X	X		X		X				X
PT/INR/PTT – not on Coumadin	X									
PT/INR/PTT – on coumadin[E K]	X	X		X		X				X
Adverse event evaluation		Continuous								
Serum β-HCG[F]	X									
ECG	X									
Radiographic tumor measurement[G K]	X				X				X	
Tissue IHC for pERK[H]	X									

A. Blood pressure measurement is required at baseline, weekly during Cycle #1 and then during Week #1 from Cycle #2 onwards. Patients will need to return to the clinic on weeks without scheduled physician evaluation for an RN evaluation or have their measurement obtained at their local physician's office or some other venue (e.g. patient-operated machine at a drug store, ambulatory blood pressure cuff, etc.) If measurements are not performed at MSKCC, patients will need to record their blood pressure and contact their study physician if it is elevated or, if normal, to provide the recordings to the study physician or nurse at their next scheduled clinic visit.
B. CBC during Week 3 of Cycle 2 and onwards can be obtained either at MSKCC or at local physician's office.

Amended:11/23/10

IRB PB

C. Comprising sodium, potassium, chloride, bicarbonate, BUN, creatinine, glucose, total bilirubin, total protein, AST, ALT, alkaline phosphatase.
D. Asymptomatic grade 3/4 hyperamylasemia and hyperlipasemia without evidence of pancreatitis were noted in 1% and 12% of patients respectively in the large phase III randomized trial of sorafenib in advanced renal cell carcinoma.
E. Because of a potential drug interaction with sorafenib, patients on chronic anticoagulation with coumadin will require periodic monitoring of their PT/INR every two weeks.
F. For women of childbearing potential (see section 8.0).
G. A CT or MRI will be obtained at baseline within two weeks of study enrollment. Repeat radiographic evaluation will be obtained after the first four weeks on study, at eight weeks, and then every eight weeks thereafter. Patients with a partial/complete response require a confirmatory scan at least four weeks after the initial scan documenting response (see Section 12.5). The same imaging modality performed at baseline (CT or MRI) will be repeated at subsequent imaging.
H. Prior archived tissue must be obtained at anytime prior to enrollment or during protocol therapy for immunohistochemical staining for phosphorylated extracellular signal-regulated kinase (pERK).
I. If pre-treatment CBC, comprehensive metabolic panel and PT/INR/PTT are drawn within seven days of beginning therapy, they do not need to be re-drawn again during Cycle #1, Week #1.
J. Cycle start date will coincide with the physician visit date.
K. Because of the potential need for blood test, CT/MRI scan, and physician visit scheduling to vary (due to both physician and patient issues), to avoid violation of, and deviation from the protocol, blood tests, CT/MRI scans, and physician visits (including physical exam, vitals, blood pressure, weight, performance status, and concomitant medications) may vary by up to one to fourteen (1-14) days from a strict 28 day schedule.

Evaluations during treatment

Please note, because of the potential need for blood test, CT/MRI scans, and physician visit scheduling to vary (due to both physician and patient issues), to avoid violation of, and deviation from, the protocol, blood tests, CT/MRI scans and physician visits (which include physical exam, vitals, blood pressure, weight measurement, performance status assessment and concomitant medications) may vary up to one to fourteen (1-14)days from a strict 28 day schedule.

- A physical exam, vitals (including blood pressure) and weight measurement, and performance status assessment on Week 1, Week 2 and Week 3 of Cycle 1 and then on Week 1 of each Cycle thereafter.
- A CBC on Week 1, Week 2 and Week 3 of Cycle 1 and then on Week 1 and Week 3 of each Cycle thereafter.
- A comprehensive metabolic panel (CMP) on Week 1 and Week 3 of Cycle 1 and then on Week 1 of each Cycle thereafter.
- An amylase/lipase level on Week 1 and Week 3 of Cycle 1 and then on Week 1 of each Cycle thereafter.

- For patients on coumadin therapy, a PT/INR/PTT on Week 1 and Week 3 of Cycle 1 and then on Week 1 of each Cycle thereafter.
- Sorafenib diary to be collected on Week 1 of each new Cycle.
- A list of concomitant medications to be obtained on Week 1 of each new Cycle.
- A repeat CT or MRI of measurable disease after the first 4 weeks on study, at eight weeks, and then every 8 weeks thereafter. The same imaging modality performed at baseline (CT or MRI) will be repeated at subsequent imaging.

Evaluation at the end of study treatment:

- A physical exam, vitals (including blood pressure) and weight measurement, and performance status assessment.
- A list of concomitant medications.
- Sorafenib diary to be collected.
- A CBC, CMP and amylase/lipase level. For patients on coumadin, a PT/INR/PTT level.

Immunohistochemistry for pERK

Phosphorylated extracellular signal-regulated kinase (pERK) determination through immunohistochemical (IHC) staining of prior paraffin stored tumor blocks or unstained slides. pERK determination may be pending at the time of protocol registration, with prior material submitted for IHC during the course of protocol therapy. The pERK status is not required for enrollment on the protocol. Studies will be performed at MSKCC.

Whenever possible, the most recent tumor biopsy/pathologic specimen prior to study enrollment will be used.

Paraffin-embedded tissue should be prepared by fixing tissues in 10% neutral buffered formalin. An optimal fixation time is 12 – 24 hours and should not exceed 48 hours. Slides should consist of a minimum of five unstained, positively charged microscope slides with 4 – 6 µm tissue sections, cut from a paraffin block. The slides should be prepared in a 37 – 42 ^{o}C water bath without gelatin. Only one 4 – 6 µm cut section is placed on each slide, opposite the frosted end, before the slides are allowed to air dry.

pERK tissue expression is assayed using a rabbit polyclonal antibody for pERK (phospho-p44/42 mitogen activated protein kinase [Thr202/Tyr204]; Cell Signaling Technology Inc, Danvers, MA). A standard secondary biotinylated anti-mouse antibody and streptavidin horseradish peroxidase are then applied for visualization.

Briefly, the procedure involves: (1) Deparaffinization and rehydration of the tissues with xylene and graded alcohols; (2) Antigen unmasking by pretreatment with sodium citrate; (3) Incubation with primary antibody or negative control sera (non-specific mouse IgG); (4) Incubation with secondary biotinylated anti-mouse antibody; (5) Incubation with streptavidin horseradish peroxidase; (6) Incubation with substrate and chromogen (DAB) (7) Counterstaining the tissue with hematoxylin-eosin; (8) Mounting with an aqueous cover slip medium and; (9) Microscopy.

A positive control cell line (derived from a gastrointestinal stromal tumor cell line that expresses pERK) is included with each assay. Each specimen is reviewed after staining for the presence of tumor cells, the localization of pERK staining to the cell nuclei or the cytoplasm, the staining intensity and the proportion of tumor cells that stain positive. pERK staining is graded semi-quantitatively using a five-point scale: 0, no staining; 1+, weak; 2+, moderate; 3+ strong and; 4+, intense.

11.0 TOXICITIES/SIDE EFFECTS

11.1 General considerations

Subjects must be carefully monitored for adverse events. This monitoring includes clinical laboratory tests. Adverse events should be assessed on a continuous basis, with the highest grade recorded according to National Cancer Institute Common Terminology Criteria for Adverse Events (NCI-CTCAE), version 3.0.[46] Adverse event should be assessed in terms of seriousness, severity, and relationship to the study drug (See Section 11.6).

11.2 General toxicity profile

For the most recent safety update, refer to the Nexavar® Prescribing Information.
The overall safety profile of sorafenib is based on 1,286 cancer patients, who received sorafenib as a single agent.[33]

Table 11.2.1 will be used to determine the "expectedness" of adverse drug reactions used for reporting of adverse events to Regulatory Agencies. It is not a complete list of adverse events reported in clinical trials.

Table 11.2.1. Adverse drug reactions in patients in multiple clinical trials.

System Organ Class	Very Common ≥ 10%	Common ≥ 1% to < 10%	Uncommon ≥ 0.1% to < 1%
Infections and infestations			Folliculitis infection
Blood and lymphatic system disorders	Lymphopenia	Leukopenia<br>Neutropenia<br>Anemia<br>Thrombocytopenia	
Immune system disorders			Hypersensitivity reactions (including skin reactions and urticaria)
Endocrine disorders			Hypothyroidism
Metabolism and nutrition disorders	Hypophosphatemia	Anorexia	Hyponatremia<br>Dehydration
Psychiatric disorders		Depression	
Nervous system disorders		Peripheral sensory neuropathy	Reversible posterior leukoencephalopathy*
Ear and labyrinth disorders		Tinnitus	
Cardiac disorders			Myocardial ischemia and infarction*<br>Congestive heart failure*
Vascular disorders	Hemorrhage (including gastrointestinal* and respiratory tract* and cerebral hemorrhage*)<br>Hypertension		Hypertensive crisis*

* Events may have a life-threatening or fatal outcome. Such events are uncommon.
**Palmar plantar erythrodysaethesia reaction

System Organ Class	Very Common ≥ 10%	Common ≥ 1% to < 10%	Uncommon ≥ 0.1% to < 1%
Respiratory, thoracic and mediastinal disorders		Hoarseness	Rhinorrhea
Gastrointestinal disorders	Diarrhea<br>Nausea<br>Vomiting	Constipation<br>Stomatitis (including dry mouth and glossodynea)<br>Dyspepsia<br>Dysphagia	Gastroesophageal reflux disease<br>Pancreatitis<br>Gastritis<br>Gastrointestinal perforation*
Hepato-biliary disorders			Increase in bilirubin and jaundice
Skin and subcutaneous tissue disorders	Rash<br>Alopecia<br>Hand-foot reaction**<br>Pruritis<br>Erythema	Dry skin<br>Dermatitis exfoliative<br>Acne<br>Skin desquamation	Eczema<br>Erythema multiforme minor
Musculoskeletal, connective tissue and bone disorders		Arthralgia<br>Myalgia	
Reproductive system and breast disorders		Erectile dysfunction	Gynecomastia
General disorders and administration site conditions	Fatigue<br>Pain (inc. mouth, abdominal, bone pain, headache and tumor pain)	Asthenia<br>Fever<br>Influenza-like illness	
Investigations	Increased amylase<br>Increased lipase	Weight decreased<br>Transient increase in transaminases	Transient increase in blood alkaline phosphatase<br>INR abnormal, prothrombin level abnormal

* Events may have a life-threatening or fatal outcome. Such events are uncommon.
**Palmar plantar erythrodysaethesia reaction

11.3 Laboratory abnormalities

The following laboratory abnormalities were observed in the randomized phase III trial of sorafenib in patients with advanced renal cell carcinoma[33]:

Elevated lipase and amylase: Elevated lipase and amylase levels were very commonly reported. Grade 3/4 lipase elevations occurred in 12% of patients in the sorafenib group compared to 7% of patients in the placebo group. Grade 3/4 amylase elevations were reported in 1% of patients in the sorafenib group compared to 3% of patients in the placebo group. Clinical pancreatitis was reported in 2 of 451 sorafenib treated patients (Grade 4) and 1 of 451 patients (Grade 2) in the placebo group.

Hypophosphatemia: Hypophosphatemia was a common laboratory finding, observed in 45% of sorafenib treated patients compared to 12% of placebo patients. Grade 3 hypophosphatemia (1–2 mg/dl) occurred in 13% of sorafenib-treated patients and 3% of patients in the placebo group. There were no cases of Grade 4 hypophosphatemia (< 1 mg/dl) reported in either sorafenib or placebo patients. The etiology of hypophosphatemia associated with sorafenib is not known.

Lymphopenia: Grade 3/4 lymphopenia was reported in 13% of sorafenib treated patients and 7% of placebo patients.

Neutropenia: Grade 3/4 neutropenia was reported in 5% of sorafenib treated patients and 2% of placebo patients.

Anemia: Anemia was observed in 44% of sorafenib-treated patients and 49% of placebo patients. Grade 3/4 anemia was reported in 2% of sorafenib-treated patients and 4% of placebo patients.

Thrombocytopenia: Thrombocytopenia was observed in 12% of sorafenib-treated patients and 5% of placebo patients. Grade 3/4 thrombocytopenia was reported in 1% of sorafenib-treated patients and 0% of placebo patients.

11.4 Special warning and precautions for use

Dermatological toxicities: Hand-foot skin reaction (palmar-plantar erythrodysaesthesia) and rash represent the most common adverse drug reactions with sorafenib. Rash and hand-foot skin reaction are usually Grade 1/2 and generally appear during the first six weeks of

treatment with sorafenib. Management of dermatologic toxicities may include topical therapies for symptomatic relief, temporary treatment interruption and/or dose modification of sorafenib, or in severe or persistent cases, permanent discontinuation of sorafenib. See Section 9.2.4 for supportive measures and Table 11.5.1 for suggested dose modifications for hand-foot reaction.

Hypertension: An increased incidence of hypertension was observed in sorafenib-treated patients. Hypertension was usually mild to moderate, occurred early in the course of treatment and was amenable to management with standard antihypertensive therapy. Blood pressure should be monitored regularly and treated, if required, in accordance with standard medical practice. In cases of severe or persistent hypertension, or hypertensive crisis despite adequate antihypertensive therapy, permanent discontinuation of sorafenib should be considered. See Table 11.5.2 for suggested dose modifications for treatment-emergent hypertension.

Hemorrhage: An increase in the risk of bleeding may occur following sorafenib administration. The incidence of severe bleeding events is uncommon. If any bleeding event necessitates medical intervention, it is recommended that permanent discontinuation of sorafenib be considered.

Wound healing complications: No formal studies of the effect of sorafenib on wound healing have been conducted. In patients undergoing major surgical procedures, temporary interruption of sorafenib therapy is recommended for precautionary reasons. There is limited clinical experience regarding the timing of reinitiation of therapy following major surgical intervention. Therefore, the decision to resume sorafenib therapy following a major surgical intervention should be based on clinical judgment of adequate wound healing.

Cardiac Ischemia and/or Infarction: In the phase 3 randomized study of sorafenib in patients with advanced renal cell carcinoma, the incidence of treatment-emergent cardiac ischemia/infarction events was higher in the sorafenib group (2.9%) compared with the placebo group (0.4%).[41] Patients with unstable coronary artery disease or recent myocardial infarction were excluded from this study. Sorafenib should be discontinued in patients who develop cardiac ischemia and/or infarction.

Gastrointestinal perforation: Gastrointestinal perforation is an uncommon event and has been reported in <1% of patients taking sorafenib.[33] In some cases, this was not associated with apparent intra-abdominal tumor. Sorafenib should be discontinued in patients with GI perforation.

Effects on ability to drive and use machines: No studies on the effects of sorafenib on the ability to drive or use machines have been performed. There is no evidence that sorafenib affects the ability to drive or operate machinery.

Patients with Hepatic Impairment: *In vitro* and *in vivo* data indicate that sorafenib is primarily metabolized by the liver. Systemic exposure and safety data were comparable in patients with Child-Pugh A and B hepatic impairment. Sorafenib has not been studied in patients with Child-Pugh C hepatic impairment. No dose adjustment is necessary when administering sorafenib to patients with Child-Pugh A and B hepatic impairment.

Patients with Renal Impairment: Sorafenib has not been studied in patients with severe renal impairment (CrCl <30 mL/min) or in patients undergoing dialysis.

Carcinogenesis, Mutagenesis, Impairment of Fertility: Carcinogenicity studies have not been performed with sorafenib. Sorafenib was clastogenic when tested in an *in vitro* mammalian cell assay (Chinese Hamster Ovary) in the presence of metabolic activation. Sorafenib was not mutagenic in the *in vitro* Ames bacterial cell assay or clastogenic in an *in vivo* mouse micronucleus assay. One intermediate in the manufacturing process, which is also present in the final drug substance (<0.15%), was positive for mutagenesis in an *in vitro* bacterial cell assay (Ames test) when tested independently. No specific studies with sorafenib have been conducted in animals to evaluate the effect on fertility. However, results from the repeat-dose toxicity studies suggest there is a potential for sorafenib to impair reproductive performance and fertility. Multiple adverse effects were observed in male and female reproductive organs, with the rat being more susceptible than mice or dogs. Typical changes in rats consisted of testicular atrophy or degeneration, degeneration of epididymis, prostate, and seminal vesicles, central necrosis of the corpora lutea and arrested follicular development. Sorafenib-related effects on the reproductive organs of rats were manifested at daily oral doses =30 mg/m^2 (approximately 0.5 times the AUC in cancer patients at the recommended human dose). Dogs showed tubular degeneration in the testes at 600 mg/m^2/day (approximately 0.3 times the AUC at the recommended human dose) and oligospermia at 1200 mg/m^2/day of sorafenib. Adequate contraception should be used during therapy and for at least 2 weeks after completing therapy. [33]

Pediatric Use: The safety and effectiveness of sorafenib in pediatric patients have not been studied. Repeat dosing of sorafenib to young and growing dogs resulted in irregular

thickening of the femoral growth plate at daily sorafenib doses =600 mg/m^2 (approximately 0.3 times the AUC at the recommended human dose), hypocellularity of the bone marrow adjoining the growth plate at 200 mg/m^2/day (approximately 0.1 times the AUC at the recommended human dose), and alterations of the dentin composition at 600 mg/m^2/day. Similar effects were not observed in adult dogs when dosed for 4 weeks or less.

Geriatric Use: In total, 32% of RCC patients treated with sorafeniab were age 65 years or older, and 4% were 75 and older. No differences in safety or efficacy were observed between older and younger patients, and other reported clinical experience has not identified differences in responses between the elderly and younger patients, but greater sensitivity of some older individuals cannot be ruled out.

Drug-Drug Interactions: Caution is recommended when administering sorafenib together with compounds that are metabolized/ eliminated predominantly by the UGT1A1 pathway (e.g. irinotecan) [33]

CYP3A4 inducers: There is no clinical information on the effect of CYP3A4 inducers on the pharmacokinetics of sorafenib. Inducers of CYP3A4 activity (e.g. rifampicin, Hypericum perforatum also known as St. John's wort, phenytoin, carbamazepine, phenobarbital, and dexamethasone) may increase metabolism of sorafenib and thus decrease sorafenib plasma concentrations.

CYP3A4 inhibitors: Ketoconazole, a potent inhibitor of CYP3A4, administered once daily for 7 days to healthy male volunteers did not alter the mean AUC of a single 50 mg dose of sorafenib. Therefore, clinical pharmacokinetic interactions of sorafenib with CYP3A4 inhibitors are unlikely.

CYP2C9 substrates: The possible effect of sorafenib on the metabolism of the CYP2C9 substrate warfarin was assessed indirectly by measuring PTINR. The mean changes from baseline in PT-INR were not higher in sorafenib patients compared to placebo patients, suggesting that sorafenib did not inhibit warfarin metabolism *in vivo*. However, patients taking warfarin should have their INR checked regularly.

CYP isoform-selective substrates: Concomitant administration of midazolam, dextromethorphan and omeprazole, which are substrates of cytochromes CYP3A4, CYP2D6 and CYP2C19, respectively, following 4 weeks of sorafenib administration did

not significantly alter the exposure of these agents. This indicates that sorafenib is neither an inhibitor nor a clinically meaningful inducer of these cytochrome P450 isoenzymes.

Combination with other anti-neoplastic agents: In clinical studies, sorafenib has been administered together with a variety of other antineoplastic agents at their commonly used dosing regimens, including gemcitabine, oxaliplatin, doxorubicin, and irinotecan. Sorafenib had no effect on the pharmacokinetics of gemcitabine or oxaliplatin. Concomitant treatment with sorafenib resulted in a 21% increase in the AUC of doxorubicin. When administered with irinotecan, whose active metabolite SN-38 is further metabolized by the UGT1A1 pathway, there was a 67-120% increase in the AUC of SN-38 and a 26-42% increase in the AUC of irinotecan. The clinical significance of these findings is unknown. However, caution is recommended when administering sorafenib with doxorubicin and with compounds that are metabolized/eliminated predominantly by the UGT1A1 pathway (e.g. irinotecan).

Warfarin: Infrequent bleeding events or elevations in the International Normalized Ratio (INR) have been reported in some patients taking warfarin while on sorafenib therapy. Patients taking warfarin concomitantly should be monitored regularly for changes in prothrombin time, INR and for clinical bleeding episodes.

11.5 Dose delay/modification

Intrapatient dose reduction will be allowed depending on the type and severity of toxicity encountered provided that criteria for patient withdrawal from study treatment have not been met. The dose levels for sorafenib are as described below:

Starting dose 0	Sorafenib 400 mg twice daily
Dose level -1	Sorafenib 400 mg daily
Dose level -2	Sorafenib 400 mg every other day

If further dose reduction is required, the patient should be taken off-protocol.

Patients who require a treatment delay of >4 consecutive weeks should also be taken off-protocol.

The following tables describe the recommended dose modifications for sorafenib-associated toxicities, including hand-foot skin reaction (Table 11.5.1), treatment-emergent hypertension (Table 11.5.2), exacerbation of baseline hypertension (Table 11.5.3) and other general hematologic and non-hematologic toxicities (Table 11.5.4).

Table 11.5.1: Suggested dose modifications for sorafenib for hand-foot skin reaction

Grade	Occurrence	Suggested Dose Modification
Grade 1<br><br>Numbness, dysesthesia, paresthesia, tingling, painless swelling, erythema or discomfort of the hands or feet which does not disrupt the patient's normal activities	Any	Promptly institute supportive measures such as topical therapy for symptomatic relief and continue sorafenib treatment.
Grade 2<br><br>Painful erythema and swelling of the hands or feet and/or discomfort affecting the patient's normal activities	First	Promptly institute supportive measures such as topical therapy for symptomatic relief and consider a decrease of sorafenib dose to 400 mg daily for a minimum of 7 days and up to 28 days.<br>• If toxicity resolves to grade 0-1 after dose reduction, increase sorafenib back to full dose of 400 mg BID.<br>• If toxicity does not resolve to grade 0-1 despite dose reduction, interrupt sorafenib treatment for a minimum of 7 days and until toxicity has resolved to grade 0-1.<br>• When resuming treatment after dose interruption, resume sorafenib at a reduced dose of 400mg daily.<br>• If toxicity is maintained at grade 0-1 at reduced dose for a minimum of 7 days and up to 28 days, increase sorafenib back to full dose of 400 mg BID
	Second or Third occurrence	As for first occurrence.<br>Upon resuming sorafenib treatment, decrease dose by one dose level (400 mg daily or 400 mg every other day).<br>Decision whether to dose re-escalate should be based on clinical judgment and patient preference.
	Fourth occurrence	Decision whether to discontinue sorafenib treatment should be made based on clinical judgment and patient preference
Grade 3<br><br>Moist desquamation, ulceration, blistering or severe pain of the hands or feet, or severe discomfort that causes the patient to be unable to work or perform activities	First occurrence	Institute supportive measures such as topical therapy for symptomatic relief and interrupt sorafenib treatment for a minimum of 7 days and until toxicity has resolved to grade 0–1<br>• When resuming treatment after dose interruption, decrease sorafenib by one dose level (400 mg daily or 400 mg every other day)<br>• If toxicity is maintained at grade 0-1 at reduced dose for a minimum of 7 days and up to 28 days, increase by one dose level (400mg BID or 400 mg daily)
	Second	As for first occurrence. Upon resuming sorafenib treatment, decrease dose by one

of daily living	occurrence	dose level (400 mg daily or 400 mg every other day).<br>Decision whether to dose re-escalate should be made based on clinical judgment and patient preference.
	Third occurrence	Decision whether to discontinue sorafenib treatment should be made based on clinical judgment and patient preference.

Table 11.5.2: Dose Modifications of Sorafenib for Hypertension

Grade (CTCAE v3.0)	Antihypertensive Therapy	Blood Pressure Monitoring	Sorafenib Dose
Grade 1	None	Routine	No change
Grade 2 (asymptomatic)	Initiate monotherapy (suggest dihydropyridine calcium-channel blocker)	Increase frequency and monitor (by health professional) every 2 days until stabilized	No change
Grade 2 (symptomatic/ persistent)<br>OR<br>Diastolic BP > 110 mm Hg<br>OR<br>Grade 3	Add agent(s):<br>Ca^{++} channel blocker (if not already used),<br>K+ channel opener (angiotensin blockers),<br>beta-blocker,<br>thiazide diuretic	Increase frequency and monitor (by health professional) every 2 days until stabilized; continue qod monitoring to stabilization after dosing restarted.	Hold* Sorafenib until symptoms resolve and diastolic BP < 100 mm/Hg.<br><br>Resume treatment at 1 dose level lower**
Grade 4			Off protocol therapy
* Patients requiring a delay of > 21 days should go off protocol therapy unless, in the treating physician's opinion, the patient may benefit from continued treatment..<br>** Patients requiring >2 dose reductions should go off protocol therapy.			
CTCAE v3.0 definitions<br>Grade 1: asymptomatic, transient (<24 hrs) increase by > 20 mmHg (diastolic) or > 150/100 if previously WNL; intervention not indicated<br>Grade 2: recurrent or persistent (>24 hrs) or symptomatic increase by >20 mmHg (diastolic) or to >150/100 if previously WNL; monotherapy may be indicated.			

Amended:11/23/10

IRB PB

Grade 3: requiring more than one drug or more intensive therapy than previously<br>Grade 4: life threatening (*e.g*., hypertensive crisis)

Table 11.5.3: Suggested dose modifications for sorafenib for exacerbation of hypertension in patients with baseline diagnosis of hypertension.

Hypertension grade	Suggested dose modification
Grade 1: Asymptomatic, transient (<24 hours) increase by >20 mmHg (diastolic) or to >150/100 mmHg if previously within normal limits (WNL)	Increase blood pressure monitoring
Grade 2: Recurrent or persistent (≥24 hours) increase by >20 mmHg (diastolic) or to >150/100 mmHg if previously WNL<br>OR<br>Grade 3: Requiring >1 drug or more intensive therapy than previously<br>BUT diastolic blood pressure <110 mmHg	Additional anti-hypertensive therapy per standard management and continue sorafenib
Grade 2 but symptomatic<br>OR<br>Diastolic blood pressure ≥110 mmHg	1. Hold sorafenib until symptoms resolve and diastolic BP <100 mmHg<br>2. Additional anti-hypertensive therapy.<br>3. Restart sorafenib at one dose level lower.*<br>4. If diastolic BP is ≥100 mmHg on anti-hypertensive therapy, decrease by another dose level.
Grade 4: Life-threatening consequences, e.g. hypertensive crisis	Discontinue sorafenib; institute supportive measures

*may be able to resume sorafenib at full-dose later if BP is <140/80 on anti-hypertensive therapy.

Table 11.5.4: Dose modifications for sorafenib hematologic and non-hematologic toxicities (other than hand-foot reaction and hypertension)

Toxicity	Grade 1	Grade 2	Grade 3*	Grade 4*
Non-hematologic	Continue at the same dose level.	Continue at the same dose level.	Withhold dose for at least 7 days until toxicity is grade ≤1, then resume treatment at one dose level lower.	Withhold dose for at least 7 days until toxicity is grade ≤1, then resume treatment at one dose level lower, or discontinue at the discretion of the principal investigator after discussion with study sponsor.
Hematologic	Continue at the same dose level.	Continue at the same dose level.	Withhold dose for at least 7 days until toxicity is grade ≤2, then resume treatment at one dose level lower.	Withhold dose for at last 7 days until toxicity is grade ≤2, then resume treatment at one dose level lower, or discontinue at the discretion of the principal investigator after discussion with study sponsor.

*Patients who develop grade 3 fever/chills, grade 3 elevation of hepatic transaminases with ALT and AST <10X ULN, grade 3 hyperlipasemia or hyperamylasemia without clinical or other evidence of pancreatitis, grade 3 leukopenia, or grade 3/grade 4 lymphopenia may continue study treatment without interruption at the discretion of the investigator.

11.6 Adverse event definitions

11.6.1 Adverse event

An adverse event is any untoward medical occurrence in a subject or clinical investigation subject administered with a pharmaceutical product. The adverse event does not necessarily have to have a causal relationship with this treatment. An adverse event can therefore be any unfavorable and unintended sign (including an abnormal laboratory finding), symptom, or disease temporally associated with the use of an investigational product, whether or not considered related to the medicinal product.

Adverse events associated with the use of a drug in humans, whether or not considered drug related, include the following:

- An adverse event occurring in the course of the use of a drug product in professional practice.
- An adverse event occurring from an overdose whether accidental or intentional.
- An adverse event occurring from drug abuse.
- An adverse event occurring from drug withdrawal.
- An adverse event where there is a reasonable possibility that the event occurred purely as a result of the subject's participation in the study (e.g. adverse event or serious adverse event due to discontinuation of anti-hypertensive drugs during wash-out phase) must also be reported as an adverse event even if it is not related to the investigational product.

11.6.2 Serious adverse event (SAE)

A serious adverse event (SAE) is any untoward medical occurrence that at any dose:

- Results in death.
- Is life-threatening.
- Requires in-patient hospitalization or prolongation of existing hospitalization.
- Results in persistent or significant disability or incapacity.
- Is a congenital anomaly or birth defect.
- Is an important medical event.

11.6.3 Adverse event documentation

All adverse events occurring after the subject has signed the informed consent must be fully recorded in the subject's case record form.

Documentation must be supported by an entry in the subject's file. A laboratory test abnormality considered clinically relevant, e.g., causing the subject to withdraw from the study, requiring treatment or causing apparent clinical manifestations, or judged relevant by the investigator, should be reported as an adverse event. Each event should be described in detail along with start and stop dates, severity, relationship to investigational product, action taken and outcome.

11.7 Reporting of serious adverse events (SAE)

Each serious adverse event must be followed up until resolution or stabilization, by submission of updated reports to the designated person. An isolated laboratory abnormality that is assigned grade 4, according to CTC definition, is not reportable as an SAE, unless the investigator assesses that the event meets standard ICH criteria for an SAE. CTC grade 4 baseline laboratory abnormalities that are part of the disease profile should not be reported as an SAE, specifically when they are allowed or not excluded by the protocol inclusion/exclusion criteria.

When required, and according to local law and regulations, serious adverse events must be reported to the Ethics Committee and Regulatory Authorities.

Serious adverse drug-related events should be reported to Bayer/Onyx. In the event of such an event, the investigator should refer to the Pharmacovigilance section of the contract for reporting procedures.
In brief, the Investigator/Sponsor may report serious adverse drug reactions (SADRs) using a Clinical Research Database (CRDB) Adverse Event report.

All reports shall be sent electronically to:

**Electronic Mailbox:**	DrugSafety.GPV.US@bayer.com
**Facsimile:**	(973) 709-2185
**Address:**<br>**Mail only**	Global Pharmacovigilance - USA<br>Bayer HealthCare Pharmaceuticals Inc.

P.O. Box 1000
Montville, NJ 07045-1000

Address: 340 Changebridge Road
FDX or UPS only Pine Brook, NJ 07058

Reports for all Bayer products can also be phoned in to the Clinical Communications Department:

Phone: 1-888-842-2937

12.0 CRITERIA FOR THERAPEUTIC RESPONSE/OUTCOME ASSESSMENT

12.1 Measurement of effect

For the purposes of this study, patients should be re-evaluated after the first cycle, i.e. four weeks, of treatment and then every two cycles or eight weeks thereafter. Confirmatory scans should also be obtained no less than four weeks following initial documentation of objective response.

12.2 Definitions

Response and progression will be evaluated in this study using standard Response Evaluation Criteria in Solid Tumors (RECIST) criteria.[45] Changes in only the largest diameter (unidimensional measurement) of the tumor lesions are used in the RECIST criteria. Note: Lesions are either measurable or non-measurable using the criteria provided below. The term "evaluable" in reference to measurability will not be used because it does not provide additional meaning or accuracy.

12.2.1 Measurable disease

Measurable lesions are defined as those that can be accurately measured in at least one dimension (longest diameter to be recorded) as ≥20 mm with conventional X-rays or ≥10 mm with CT or MRI scan. All tumor measurements must be recorded in millimeters (or decimal fractions of centimeters).

Tumor lesions within a prior radiation field are considered measurable as long as disease progression has been documented in these lesions after prior radiotherapy.

12.2.2 Non-measurable disease

All other lesions (or sites of disease), including small lesions (longest diameter <20 mm using X-ray or <10 mm using CT or MRI scan), are considered non-measurable disease. Bone lesions, leptomeningeal disease, ascites, pleural/pericardial effusions, lymphangitis cutis/pulmonis, inflammatory breast disease, abdominal masses (not followed by CT or MRI), and cystic lesions are all non-measurable.

12.2.3 Target lesions

All measurable lesions up to a maximum of five lesions per organ and 10 lesions in total, representative of all involved organs, should be identified as target lesions and recorded and measured at baseline. Target lesions should be selected on the basis of their size (lesions with the longest diameter) and their suitability for accurate repeated measurements (either by imaging techniques or clinically). A sum of the longest diameter (LD) for all target lesions will be calculated and reported as the baseline sum LD. The baseline sum LD will be used as reference by which to characterize the objective tumor response.

12.2.4 Non-target lesions

All other lesions (or sites of disease) should be identified as non-target lesions and should also be recorded at baseline. Non-target lesions include measurable lesions that exceed the maximum numbers per organ or total of all involved organs as well as non-measurable lesions. Measurements of these lesions are not required but the presence or absence of each should be noted throughout follow-up.

12.3 Guidelines for evaluation of measurable disease

All measurements should be taken and recorded in metric notation using a ruler or calipers. All baseline evaluations should be performed within four weeks before the beginning of the treatment.

The same method of assessment and the same technique should be used to characterize each identified and reported lesion at baseline and during follow-up. Imaging-based evaluation is

preferred to evaluation by clinical examination when both methods have been used to assess the antitumor effect of a treatment.

Clinical lesions. Clinical lesions are not considered measurable.

Chest X-ray. Lesions on chest x-ray are acceptable as measurable lesions when they are clearly defined and surrounded by aerated lung. However, CT is preferable.

Conventional CT and MRI. These techniques should be performed with cuts of ≤10 mm in slice thickness contiguously. Spiral CT should be performed using a 5 mm contiguous reconstruction algorithm. This applies to tumors of the chest, abdomen, and pelvis. Head and neck tumors and those of extremities usually require specific protocols.

Ultrasound (U/S). U/S should not be used to measure tumor lesions. It is, however, a possible alternative to clinical measurements of superficial palpable lymph nodes, subcutaneous lesions and thyroid nodules. U/S might also be useful to confirm the complete disappearance of superficial lesions usually assessed by clinical examination.

Endoscopy/laparoscopy. The utilization of these techniques for objective tumor evaluation has not yet been fully and widely validated. Their uses in this specific context require sophisticated equipment and a high level of expertise. Therefore, the utilization of such techniques for objective tumor response is not permitted. However, such techniques can be useful to confirm pathological complete response when biopsies are obtained.

Tumor markers. There are no validated tumor markers for the assessment of esophageal and GE junction cancers. As such, they cannot be used to assess response.

12.4 Response Criteria

12.4.1 Evaluation of target lesions

Complete Response (CR):	Disappearance of all target lesions
Partial Response (PR):	≥30% decrease in the sum of the longest diameter (LD) of target lesions, taking as reference the baseline sum LD

Progressive Disease (PD):	≥20% increase in the sum of the LD of target lesions, taking as reference the smallest sum LD recorded since the treatment started or the appearance of one or more new lesions
Stable Disease (SD):	Neither sufficient shrinkage to qualify for PR nor sufficient increase to qualify for PD, taking as reference the smallest sum LD since the treatment started

12.4.2 Evaluation of non-target lesions

Complete Response (CR):	Disappearance of all non-target lesions
Incomplete Response/ Stable Disease (SD):	Persistence of one or more non-target lesion(s)
Progressive Disease (PD):	Appearance of one or more new lesions and/or unequivocal progression of existing non-target lesions

Although a clear progression of "non-target" lesions only is exceptional, in such circumstances the opinion of the treating physician should prevail, and the progression status should be confirmed at a later time.

12.4.3 Evaluation of best overall response

The best overall response is the best response recorded from the start of the treatment until disease progression/recurrence (taking as reference for progressive disease the smallest measurements recorded since the treatment started). The patient's best response assignment will depend on the achievement of both measurement and confirmation criteria (see section 12.4.1).

Target Lesions	Non-Target Lesions	New Lesions	Overall Response
CR	CR	No	CR
CR	Incomplete response/SD	No	PR

PR	Non-PD	No	PR
SD	Non-PD	No	SD
PD	Any	Yes or No	PD
Any	PD	Yes or No	PD
Any	Any	Yes	PD

Note:
1. Patients with a global deterioration of health status requiring discontinuation of treatment without objective evidence of disease progression at that time should be classified as having "symptomatic deterioration." Every effort should be made to document the objective progression, even after discontinuation of treatment.
2. In some circumstances, it may be difficult to distinguish residual disease from normal tissue. When the evaluation of complete response depends on this determination, it is recommended that the residual lesion be investigated (fine needle aspirate/biopsy) before confirming the complete response status.

12.5 Confirmatory measurement/duration of response

12.5.1 Confirmation

To be assigned a status of PR or CR, changes in tumor measurements must be confirmed by repeat assessments that should be performed no less than 4 weeks after the criteria for response are first met. In the case of SD, follow-up measurements must have met the SD criteria at least once after study entry at a minimum interval of 4 weeks.

12.5.2 Duration of overall response

The duration of overall response is measured from the time measurement criteria are met for CR or PR (whichever is first recorded) until the first date that recurrent or progressive disease is objectively documented (taking as reference for progressive disease the smallest measurements recorded since the treatment started).

The duration of overall CR is measured from the time measurement criteria are first met for CR until the first date that recurrent disease is objectively documented.

12.5.3 Duration of stable disease

Stable disease is measured from the start of the treatment until the criteria for progression are met, taking as reference the smallest measurements recorded since the treatment started.

12.6 Progression-free survival

Progression-free survival (PFS) is defined as the duration of time from start of treatment to time of progression or death from any cause. Patients who are taken off-study because of drug-related toxicity or other adverse event but who are alive and have stable or responding disease at the time of trial discontinuation will be not be censored and will be counted in the survival analyses.

13.0 CRITERIA FOR REMOVAL FROM STUDY

Treatment will continue indefinitely until one of the following criteria apply:

- Disease progression (as determined in Section 12.4.1);
- Intercurrent illness that prevents further drug administration;
- Unacceptable adverse events (as determined in Section 11);
- A treatment delay of >4 consecutive weeks;
- Patient's death;
- Patient's decision to withdraw informed consent;
- General or specific changes in the patient's condition that renders the patient unsuitable for further treatment;
- Patient becomes pregnant;
- Non-compliance by the patient with protocol requirements;
- Patient is lost to follow-up. If the patient fails to return for follow-up evaluations, every effort should be made to re-establish contact and to determine patient outcome.

14.0 BIOSTATISTICS

This is a single institution, open-label, non-randomized, single-arm phase II evaluation. The primary objective of this study is to determine the 2-month progression free survival (PFS) for patients with advanced or recurrent esophageal and gastroesophageal (GE) junction cancer who are treated with sorafenib 400 mg twice daily. PFS is defined in Section 12.6.

The historical control 2 month PFS for patients with untreated advanced esophageal and GE junction cancer is approximately 50%[29] . Using an exact binomial single stage design we require 35 patients to differentiate between 2-month PFS of 50% and 72% with type I and II error rates of 10% each. If 22 or more patients are alive and progression free at 2 months, the regimen is declared promising. With a total of 35 patients, we also have approximately 89% power to detect an improvement in the response rate from 5% to 18% with a type I error rate of 10%.

If at any time there are 14 or more deaths or progressions before 2 months then the trial will close as it is mathematically impossible to achieve the desired endpoint.

We assume an accrual rate of 1-2 patients per month for a study duration of 18-35 months.

Secondary endpoints involve an assessment of the overall response rate (complete and partial responses), as determined by RECIST criteria. Toxicities will also be assessed and tabulated. While the small sample size precludes any definitive conclusions about differential responses based on the squamous cell carcinoma and adenocarcinoma histologies, any differences observed between the two histologies may be exploratory and hypothesis-generating. Finally, tumor tissue will undergo immunohistochemical staining for phosphorylated extracellular signal-regulated kinase (pERK). Any differential response based on different levels of pERK tumor expression will similarly be hypothesis-generating. Fisher's exact test will be employed to investigate these exploratory analyses.

15.0 RESEARCH PARTICIPANT REGISTRATION AND RANDOMIZATION PROCEDURES

15.1 Research participant registration

Confirm eligibility as defined in the section entitled Criteria for Patient/Subject Eligibility.

Obtain written informed consent, by following procedures defined in section entitled Informed Consent Procedures.

All particpants must be registered through the Protocol Participant Registration (PPR) Office at Memorial Sloan-Kettering Cancer Center. PPR is available Monday through Friday from 8:30am - 5:30pm at (646) 735-8000. The PPR fax numbers are (646) 735-0008 and (646) 735-0003. Registrations can be phoned in or faxed. The completed signature page of the informed consent form, the completed signature page of the Research Authorization and a completed Eligibility Checklist must be faxed to PPR.

During the registration process registering individuals will be required to answer specific eligibility questions and provide the following information:

Registering Individual	[Last, First Name]
Research Authorization	[Date]
MSKCC IRB Protocol#	
Attending of Record (if applicable)	[Last, First Name]
Consenting Professional	[Last, First Name]
Informed Consent Date	
Participant's Full Name	[Last, First Name]
Participant MRN	

16.0 DATA MANAGEMENT ISSUES

A Research Study Assistant (RSA) will be assigned to the study. The responsibilities of the RSA include project compliance, data collection, abstraction and entry, data reporting, regulatory monitoring, problem resolution and prioritization, and coordinate the activities of the protocol study team.

Amended:11/23/10

IRB PB

The data collected for this study will be entered into a secure database. Source documentation will be available to support the computerized patient record.

16.1 Quality assurance

Weekly registration reports will be generated to monitor patient accruals and completeness of registration data. Routine data quality reports will be generated to assess missing data and inconsistencies. Accrual rates and extent and accuracy of evaluations and follow-up will be monitored periodically throughout the study period and potential problems will be brought to the attention of the study team for discussion and action

Random-sample data quality and protocol compliance audits will be conducted by the study team, at a minimum of two times per year, more frequently if indicated.

16.2 Data and safety monitoring

The Data and Safety Monitoring (DSM) Plans at Memorial Sloan-Kettering Cancer Center were approved by the National Cancer Institute in September 2001. The plans address the new policies set forth by the NCI in the document entitled "Policy of the National Cancer Institute for Data and Safety Monitoring of Clinical Trials" which can be found at: http://cancertrials.nci.nih.gov/researchers/dsm/index.html. The DSM Plans at MSKCC were established and are monitored by the Office of Clinical Research. The MSKCC Data and Safety Monitoring Plans can be found on the MSKCC Intranet at: *http://mskweb2.mskcc.org/irb/index.htm*

There are several different mechanisms by which clinical trials are monitored for data, safety and quality. There are institutional processes in place for quality assurance (e.g., protocol monitoring, compliance and data verification audits, therapeutic response, and staff education on clinical research QA) and departmental procedures for quality control, plus there are two institutional committees that are responsible for monitoring the activities of our clinical trials programs. The committees: *Data and Safety Monitoring Committee (DSMC)* for Phase I and II clinical trials, and the *Data and Safety Monitoring Board (DSMB)* for Phase III clinical trials, report to the Center's Research Council and Institutional Review Board.

During the protocol development and review process, each protocol will be assessed for its level of risk and degree of monitoring required. Every type of protocol (e.g., NIH sponsored, in-house sponsored, industrial sponsored, NCI cooperative group, etc.) will be addressed and the monitoring procedures will be established at the time of protocol activation

17.0 PROTECTION OF HUMAN SUBJECTS

Inclusion of women and minorities

MSKCC has filed forms HHS 441 (civil rights), HHS 641 (handicapped individual), HHS 639-A (sex discrimination) and HHS 680 (age discrimination); we also take due notice of the NIH/ADAMHA policy concerning inclusion of women and minorities in clinical research populations. Patients of all races, both males and female, will be enrolled into the protocol. In the New York metropolitan area, there is a high proportion of minority patients (e.g. African-American, Hispanic). In general, about 15% of patients at MSKCC are members of minority ethnic groups. We will actively try to recruit minority patients to this protocol.

Exclusion of children and lactating or pregnant women

Children are excluded from this protocol because there is insufficient data to determine the safety of sorafenib in children. In addition, the incidence of esophageal cancer in children is very low. Similarly, lactating and pregnant women are excluded because of the potential teratogenicity and embryotoxicity of sorafenib.

Benefits

It is possible that this treatment will result in shrinkage of tumors or in stabilization of an otherwise progressive disease. It is not known, of course, whether these or any other favorable events will occur. It is not known if this treatment will affect the overall survival of patients.

Costs

The patient will be responsible for the cost of standard medical care, including all drug administration fees and all hospitalizations, even for complications of treatment. Sorafenib will be provided to the patient without charge. Correlative tests will be performed without charge to the patient.

Incentives

No incentives will be offered to patients/subjects to participate in this study.

Amended:11/23/10

IRB PB

Alternatives

For patients with metastatic or recurrent esophageal cancer, standard therapy includes standard palliative chemotherapy, radiation or possibly surgery. The patient may also be eligible to participate in other investigational studies. Supportive care is also an option.

Confidentiality

Every effort will be made to maintain patient confidentiality. Research and hospital records are confidential. Patient's name or any other personally identifying information will not be used in reports or publications arising from this study. The Food and Drug Administration or other authorized agencies (e.g. qualified monitors from MSKCC, Bayer Pharmaceuticals) may review patient record and pathology slides, as required. The Investigator should contain Bayer Pharmaceuticals immediately if contacted by a regulatory agency about an inspection at his/her center.

Institutional Review Board
Before study initiation, the Investigator must have written and dated approval/favorable opinion from the IRB for the protocol form, informed consent, patient recruitment materials/process (e.g. advertisements) and any other written information to be provided to patients. The Investigator should also provide the IRB with reports, updates and other information (e.g. Safety Updates, Amendments, Administrative Letters) according to regulatory requirements or institutional procedures. Copies of the initial IRB approval as well as annual re-approvals must be submitted to the study sponsor, Bayer Pharmaceuticals.

17.1 Privacy

MSKCC's Privacy Office may allow the use and disclosure of protected health information pursuant to a completed and signed Research Authorization form. The use and disclosure of protected health information will be limited to the individuals described in the Research Authorization form. A Research Authorization form must be completed by the Principal Investigator and approved by the IRB and Privacy Board.

17.2 Serious Adverse Event (SAE) Reporting

Any SAE must be reported to the IRB as soon as possible but no later than five calendar days. The IRB requires a Clinical Research Database (CRDB) AE report to be delivered to the Institutional SAE Manager (307 East 63rd Street, 1st Floor) containing the following information:

Fields populated from the CRDB:

- Subject's name (generate the report with only initials if it will be sent outside of MSKCC)
- Medical record number
- Disease/histology (if applicable)
- Protocol number and title

Data needing to be entered:

- The date the adverse event occurred
- The adverse event
- Relationship of the adverse event to the treatment (drug, device, or intervention)
- If the AE was expected
- The severity of the AE
- The intervention
- Detailed text that includes the following information:
  - A explanation of how the AE was handled
  - A description of the subject's condition
  - Indication if the subject remains on the study
  - If an amendment will need to be made to the protocol and/or consent form

The PI's signature and the date it was signed are required on the completed report.

For IND/IDE protocols:
The CRDB AE report should be completed as above and the FDA assigned IND/IDE number written at the top of the report. The report will be forwarded to the FDA by the Institutional SAE Manager through the IND Office.

For industrial protocols:
Serious adverse drug-related events should be reported to Bayer/Onyx. In such an event, the investigator should refer to the Pharmacovigilance section of the contract for reporting procedures.

Any safety information exchange between Bayer/Onyx and investigator shall be sent electronically (electronic mailbox) or via facsimile to the attention of the contacts listed below:

**Electronic Mailbox:**	DrugSafety.GPV.US@bayer.com
**Facsimile:**	(973) 709-2185
**Address:** **Mail only**	Global Pharmacovigilance - USA<br>Bayer HealthCare Pharmaceuticals Inc.<br>P.O. Box 1000<br>Montville, NJ 07045-1000
**Address:** **FDX or UPS only**	340 Changebridge Road<br>Pine Brook, NJ 07058

Reports for all Bayer products can also be phoned in to the Clinical Communications Dept:

**Phone:**	1-888-842-2937

18.0 INFORMED CONSENT PROCEDURES

All patients will be required to sign an Informed Consent, which meets the requirements of the Code of Federal Regulations 21 CFR 50.25 (Elements of Informed Consent) and the IRB of this center.

Before any protocol-specific procedures are carried out, investigators and/or designated staff (see section 15.1) will fully explain the details of the protocol, study procedures and the aspects of patient privacy concerning research-specific information. In addition to signing the IRB Informed Consent, all patients must sign the Research Authorization component of the Informed Consent form. The Research Authorization requires a separate set of signatures from the patient and the research staff concerning the subject. Three copies of the Informed Consent will be signed. One copy will be part of the patient's medical record, one copy will be placed in the research file and each patient will receive a copy of the Informed consent.

18.1 Research authorization

Procedures for obtaining Research Authorization: Before any protocol-specific procedures are carried out, investigators and/or designated staff will fully explain the details of the protocol, study procedures, and the aspects of patient privacy concerning research specific information. In addition to signing the IRB Informed Consent, all patients must sign the Research Authorization component of the informed consent form. The Research Authorization requires a separate set of signatures from the patient. The original signed documents will become part of the patient's medical record, and each patient will receive a copy of the signed documents.

19.0 REFERENCE(S)

1. Jemal A, Siegel R, Ward E, et al: Cancer statistics, 2007. CA Cancer J Clin 57:43-66, 2007
2. Crew KD, Neugut AI: Epidemiology of upper gastrointestinal malignancies. Semin Oncol 31:450-64, 2004
3. Devesa SS, Fraumeni JF, Jr.: The rising incidence of gastric cardia cancer. J Natl Cancer Inst 91:747-9, 1999
4. Orringer MB, Marshall B, Iannettoni MD: Transhiatal esophagectomy: clinical experience and refinements. Ann Surg 230:392-400; discussion 400-3, 1999
5. Urba SG, Orringer MB, Turrisi A, et al: Randomized trial of preoperative chemoradiation versus surgery alone in patients with locoregional esophageal carcinoma. J Clin Oncol 19:305-13, 2001
6. Walsh TN, Noonan N, Hollywood D, et al: A comparison of multimodal therapy and surgery for esophageal adenocarcinoma. N Engl J Med 335:462-7, 1996
7. Enzinger PC, Mayer RJ: Esophageal cancer. N Engl J Med 349:2241-52, 2003
8. Van Cutsem E, Moiseyenko VM, Tjulandin S, et al: Phase III study of docetaxel and cisplatin plus fluorouracil compared with cisplatin and fluorouracil as first-line therapy for advanced gastric cancer: a report of the V325 Study Group. J Clin Oncol 24:4991-7, 2006
9. Folkman J: Angiogenesis and angiogenesis inhibition: an overview. Exs 79:1-8, 1997
10. Weidner N, Semple JP, Welch WR, et al: Tumor angiogenesis and metastasis--correlation in invasive breast carcinoma. N Engl J Med 324:1-8, 1991

11. Weidner N, Folkman J, Pozza F, et al: Tumor angiogenesis: a new significant and independent prognostic indicator in early-stage breast carcinoma. J Natl Cancer Inst 84:1875-87, 1992
12. Ferrara N, Davis-Smyth T: The biology of vascular endothelial growth factor. Endocr Rev 18:4-25, 1997
13. Hurwitz H, Fehrenbacher L, Novotny W, et al: Bevacizumab plus irinotecan, fluorouracil, and leucovorin for metastatic colorectal cancer. N Engl J Med 350:2335-42, 2004
14. Sandler A, Gray R, Perry MC, et al: Paclitaxel-carboplatin alone or with bevacizumab for non-small-cell lung cancer. N Engl J Med 355:2542-50, 2006
15. Miller K: E2100: a randomized phase III trial of paclitaxel versus paclitaxel plus bevacizumab as first-line therapy for locally recurrent or metastatic breast cancer., Proc Amer Soc Clin Oncol, 2005
16. Shah MA, Ramanathan RK, Ilson DH, et al: Multicenter phase II study of irinotecan, cisplatin, and bevacizumab in patients with metastatic gastric or gastroesophageal junction adenocarcinoma. J Clin Oncol 24:5201-6, 2006
17. Bang Y, Kang Y, Kang W, et al: Sunitinib as second-line treatment for advanced gastric cancer: preliminary results from a phase II study. J Clin Oncol 25, 2007 (abstr 4603)
18. Kleespies A, Guba M, Jauch KW, et al: Vascular endothelial growth factor in esophageal cancer. J Surg Oncol 87:95-104, 2004
19. Mobius C, Stein HJ, Becker I, et al: The 'angiogenic switch' in the progression from Barrett's metaplasia to esophageal adenocarcinoma. Eur J Surg Oncol 29:890-4, 2003
20. Vallbohmer D, Peters JH, Kuramochi H, et al: Molecular determinants in targeted therapy for esophageal adenocarcinoma. Arch Surg 141:476-81; discussion 481-2, 2006
21. Kleespies A, Bruns CJ, Jauch KW: Clinical significance of VEGF-A, -C and -D expression in esophageal malignancies. Onkologie 28:281-8, 2005
22. Saad RS, El-Gohary Y, Memari E, et al: Endoglin (CD105) and vascular endothelial growth factor as prognostic markers in esophageal adenocarcinoma. Hum Pathol 36:955-61, 2005
23. Beeram M, Patnaik A, Rowinsky EK: Raf: a strategic target for therapeutic development against cancer. J Clin Oncol 23:6771-90, 2005
24. Khosravi-Far R, Der CJ: The Ras signal transduction pathway. Cancer Metastasis Rev 13:67-89, 1994
25. Campbell SL, Khosravi-Far R, Rossman KL, et al: Increasing complexity of Ras signaling. Oncogene 17:1395-413, 1998
26. Chong H, Vikis HG, Guan KL: Mechanisms of regulating the Raf kinase family. Cell Signal 15:463-9, 2003

27. Zhang J, Zhi H, Zhou C, et al: Up-regulation of fibronectin in oesophageal squamous cell carcinoma is associated with activation of the Erk pathway. J Pathol 207:402-9, 2005
28. Karamouzis MV, Grandis JR, Argiris A: Therapies directed against epidermal growth factor receptor in aerodigestive carcinomas. Jama 298:70-82, 2007
29. Tew W, Shah M, Schwartz G, et al: Phase II trial of erlotonib for second-line treatment in advanced esophageal cancer. Proc GI ASCO, 2005 (abstr 5).
30. Janmaat ML, Gallegos-Ruiz MI, Rodriguez JA, et al: Predictive factors for outcome in a phase II study of gefitinib in second-line treatment of advanced esophageal cancer patients. J Clin Oncol 24:1612-9, 2006
31. Wilhelm SM, Carter C, Tang L, et al: BAY 43-9006 exhibits broad spectrum oral antitumor activity and targets the RAF/MEK/ERK pathway and receptor tyrosine kinases involved in tumor progression and angiogenesis. Cancer Res 64:7099-109, 2004
32. Hilger RA, Scheulen ME, Strumberg D: The Ras-Raf-MEK-ERK pathway in the treatment of cancer. Onkologie 25:511-8, 2002
33. Sorafenib Investigator's Brochure, 2006
34. Strumberg D, Richly H, Hilger RA, et al: Phase I clinical and pharmacokinetic study of the Novel Raf kinase and vascular endothelial growth factor receptor inhibitor BAY 43-9006 in patients with advanced refractory solid tumors. J Clin Oncol 23:965-72, 2005
35. Awada A, Hendlisz A, Gil T, et al: Phase I safety and pharmacokinetics of BAY 43-9006 administered for 21 days on/7 days off in patients with advanced, refractory solid tumours. Br J Cancer 92:1855-61, 2005
36. Clark JW, Eder JP, Ryan D, et al: Safety and pharmacokinetics of the dual action Raf kinase and vascular endothelial growth factor receptor inhibitor, BAY 43-9006, in patients with advanced, refractory solid tumors. Clin Cancer Res 11:5472-80, 2005
37. Moore M, Hirte HW, Siu L, et al: Phase I study to determine the safety and pharmacokinetics of the novel Raf kinase and VEGFR inhibitor BAY 43-9006, administered for 28 days on/7 days off in patients with advanced, refractory solid tumors. Ann Oncol 16:1688-94, 2005
38. Richly H, Kupsch P, Passage K, et al: A phase I clinical and pharmacokinetic study of the Raf kinase inhibitor (RKI) BAY 43-9006 administered in combination with doxorubicin in patients with solid tumors. Int J Clin Pharmacol Ther 41:620-1, 2003
39. Siu LL, Awada A, Takimoto CH, et al: Phase I trial of sorafenib and gemcitabine in advanced solid tumors with an expanded cohort in advanced pancreatic cancer. Clin Cancer Res 12:144-51, 2006
40. Ratain MJ, Eisen T, Stadler WM, et al: Phase II placebo-controlled randomized discontinuation trial of sorafenib in patients with metastatic renal cell carcinoma. J Clin Oncol 24:2505-12, 2006

41. Escudier B, Eisen T, Stadler WM, et al: Sorafenib in advanced clear-cell renal-cell carcinoma. N Engl J Med 356:125-34, 2007
42. Bukowski R, Eisen T, Szczylik C, et al: Final results of the randomized phase III trial of sorafenib in advanced renal cell carcinoma: Survival and biomarker analysis. J Clin Oncol 25, 2007 (abstr 5023)
43. Llovet J, Ricci S, Mazzaferro V, et al: Sorafenib improves survival in advanced Hepatocellular Carcinoma (HCC): Results of a Phase III randomized placebo-controlled trial (SHARP trial). J Clin Oncol 25, 2007 (abstr LBA1)
44. Abou-Alfa GK, Schwartz L, Ricci S, et al: Phase II study of sorafenib in patients with advanced hepatocellular carcinoma. J Clin Oncol 24:4293-300, 2006
45. Therasse P, Arbuck SG, Eisenhauer EA, et al: New guidelines to evaluate the response to treatment in solid tumors. European Organization for Research and Treatment of Cancer, National Cancer Institute of the United States, National Cancer Institute of Canada. J Natl Cancer Inst 92:205-16, 2000
46. Trotti A, Colevas AD, Setser A, et al: CTCAE v3.0: development of a comprehensive grading system for the adverse effects of cancer treatment. Semin Radiat Oncol 13:176-81, 2003
47. Simon R: Optimal two-stage designs for phase II clinical trials. Control Clin Trials 10:1-10, 1989
48. Giacconne G. The potential of antiangiogenic therapy in non-small cell lung cancer. Clin Cancer Res 13: 1961-1970.
49. Cabebe E, Wakele H. Role of anti-angiogenesis agents in treating NSCLC: focus on bevacizumab and VEGR tyrosine kinase inhibitors. Curr Treat Opin Oncol 8:15-27; 2007.50.
50. Choong NW, Cohen EE, Kozloff MF et al. Phase II trial of sunitinib in patients with recurrent and or metastatic head and neck cancer. J Clin Oncol 26 (S): Abstract 6064.
51. Feinstein TM, Raez LE, Rajasnenan KK et al. Pemetrexed and bevacizumab in patients with recurrent or metastatic head and neck squamous cell carcinoma: updated results of a phase II trial. J Clin Oncol 26 (S): Abstract 6069.
52. Kies MS, Gibson MK, Kim SW et al. Cetuximab and bevacizumab in patients with recurrent or metastatic head and neck squamous cell carcinoma: an interim analysis. J Clin Oncol 26 (S): Abstract 6072.
53. Dragovich T, McCoy SM, Fenoglio-Preiser et al. Phase II trial of erlotinib in gastroesophageal junction and gastric adenocarcinomas: SWOG 0127. J Clin Oncol 24:4922-4927; 2006.

54. Ferry DR, Anderson M, Beddard K et al. A phase II study of gefitinib monotherapy in advanced esophageal adenocarcinoma: evidence of gene expression, cellular, and clinical response. Clin Cancer Res 13: 5869-5875; 2007.
55. Janmaat ML, Gallegos-Ruis MI, Rodriguez JA et al. Predictive factors for outcome in phase II study of gefitinib in second-line treatment of advanced esophageal cancer patients. J Clin Oncol 24:1612-1619; 2006.

20.0 APPENDICES

Appendix A: Sorafenib Medication Diary
